# Transcriptional specialization of human dendritic cell subsets in response to microbial vaccines

**DOI:** 10.1038/ncomms6283

**Published:** 2014-10-22

**Authors:** Romain Banchereau, Nicole Baldwin, Alma-Martina Cepika, Shruti Athale, Yaming Xue, Chun I Yu, Patrick Metang, Abhilasha Cheruku, Isabelle Berthier, Ingrid Gayet, Yuanyuan Wang, Marina Ohouo, LuAnn Snipes, Hui Xu, Gerlinde Obermoser, Derek Blankenship, Sangkon Oh, Octavio Ramilo, Damien Chaussabel, Jacques Banchereau, Karolina Palucka, Virginia Pascual

**Affiliations:** 1 Baylor Institute for Immunology Research, 3434 Live Oak Street, Dallas, Texas 75204, USA; 2 Nationwide Children’s Hospital, 700 Children’s Drive, Columbus, Ohio 43205, USA; 3 Benaroya Research Institute, 1201 9th Avenue, Seattle, Washington 98101, USA; 4 Sidra Medical and Research Center, Doha, Qatar; 5 Jackson Laboratory for Genomic Medicine, 263 Farmington Ave., Farmington, Connecticut 06030, USA

## Abstract

The mechanisms by which microbial vaccines interact with human APCs remain elusive. Herein, we describe the transcriptional programs induced in human DCs by pathogens, innate receptor ligands and vaccines. Exposure of DCs to influenza, *Salmonella enterica* and *Staphylococcus aureus* allows us to build a modular framework containing 204 transcript clusters. We use this framework to characterize the responses of human monocytes, monocyte-derived DCs and blood DC subsets to 13 vaccines. Different vaccines induce distinct transcriptional programs based on pathogen type, adjuvant formulation and APC targeted. Fluzone, Pneumovax and Gardasil, respectively, activate monocyte-derived DCs, monocytes and CD1c+ blood DCs, highlighting APC specialization in response to vaccines. Finally, the blood signatures from individuals vaccinated with Fluzone or infected with influenza reveal a signature of adaptive immunity activation following vaccination and symptomatic infections, but not asymptomatic infections. These data, offered with a web interface, may guide the development of improved vaccines.

## Abstract (web summary)

The effects of vaccines on human dendritic cells (DCs) are incompletely understood. Here the authors build a transcriptional modular network based on *in vitro* infection of human DCs and apply it for the characterization of the DC response to 13 different vaccines.

## Graphical abstract

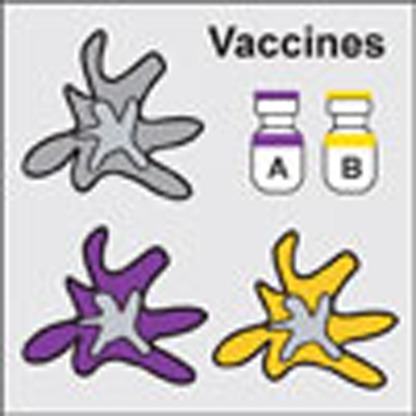

Vaccination, the most successful preventive measure against infectious diseases, relies on the presentation of microbial antigens to the adaptive immune system by antigen-presenting cells (APCs). This process leads to the generation of protective immune responses mediated by T and B cells^1^. Many vaccines that have been developed empirically have proven effective against life-threatening infections, including poliomyelitis, measles, pertussis, smallpox or mumps. Nevertheless, the lack of efficacious vaccines against modern pandemics such as human immunodeficiency virus, tuberculosis or malaria underscores the need to better understand the immunological mechanisms involved in vaccination.

Vaccination relies on dendritic cells (DCs) as demonstrated by the loss of sensitization of the immune system to cell-associated antigen in DC-depleted mice^2^. Multiple DC subsets have been identified in both mice and humans in the blood, skin, lymphoid and mucosal tissues^3^,^4^,^5^,^6^,^7^,^8^,^9^,^10^,^11^,^12^,^13^. In human blood, these include CD1c+ DCs, which are equipped with a wide range of pattern recognition receptors (PRRs) and are good inducers of both CD8+ and CD4+ T-cell responses; CD141+ DCs, which efficiently cross-present necrotic and non-self antigens to CD8+ T cells; plasmacytoid DCs, which secrete large amounts of type-I interferon (IFN) on challenge with viruses and nucleic acids; and monocyte-derived inflammatory DCs, which can be found in tissues under certain inflammatory conditions^14^,^15^,^16^. Distinct DC subsets express different PRRs, including Toll-like receptors (TLRs), C-type lectin receptors, nucleotide-binding oligomerization domain-like receptors and helicases^17^,^18^,^19^,^20^,^21^,^22^. The role of these subsets in mounting specific immune responses to different vaccines remains mostly unexplored.

DCs respond to pathogenic signals by transcribing various sets of genes that interact in a complex fashion, as highlighted by the antagonism between antiviral and antibacterial pathways^23^,^24^,^25^,^26^. Systems biology approaches such as genome-wide microarrays provide molecular snapshots of perturbed pathways^27^,^28^. Previous studies in blood leukocytes and tissues have demonstrated the applicability of microarrays to characterize the molecular mechanisms involved in infection, cancer, autoimmunity^29^,^30^,^31^,^32^,^33^,^34^,^35^,^36^,^37^ and vaccination^38^,^39^,^40^, leading to diagnostic and therapeutic advances. DC transcriptional programs at steady state or in response to various pathogen-associated molecular patterns have been examined *in vitro* and *in vivo*, using combinations of microarrays and small interfering RNA in mammalian cells^41^,^42^,^43^,^44^,^45^. The interpretation of these complex data sets has been simplified by dimension-reducing approaches such as gene co-expression module frameworks^46^. Concurrently, systems vaccinology has emerged^47^,^48^,^49^,^50^ as a discipline that leverages systems biology approaches to study the mechanisms of action of vaccines, and identify immunological correlates of protection. Despite these efforts, a significant gap of knowledge remains in the understanding of how vaccines interact with human DC subsets, and how this leads to the development of protective immunity.

Herein, we used a multi-step approach to understand human DC responses to vaccine challenge *in vitro*. We built a novel transcriptional modular framework from *in vitro*-generated DCs stimulated with pathogens and validated this framework in an independent data set of DCs stimulated with microbial components and inflammatory cytokines. We then applied this framework to characterize the early response of human DCs and their precursors to 13 microbial vaccines, thereby identifying common and specific transcriptional networks to each pathogenic challenge.

## Results

### DC transcriptional responses to pathogens

**Figure 1:**
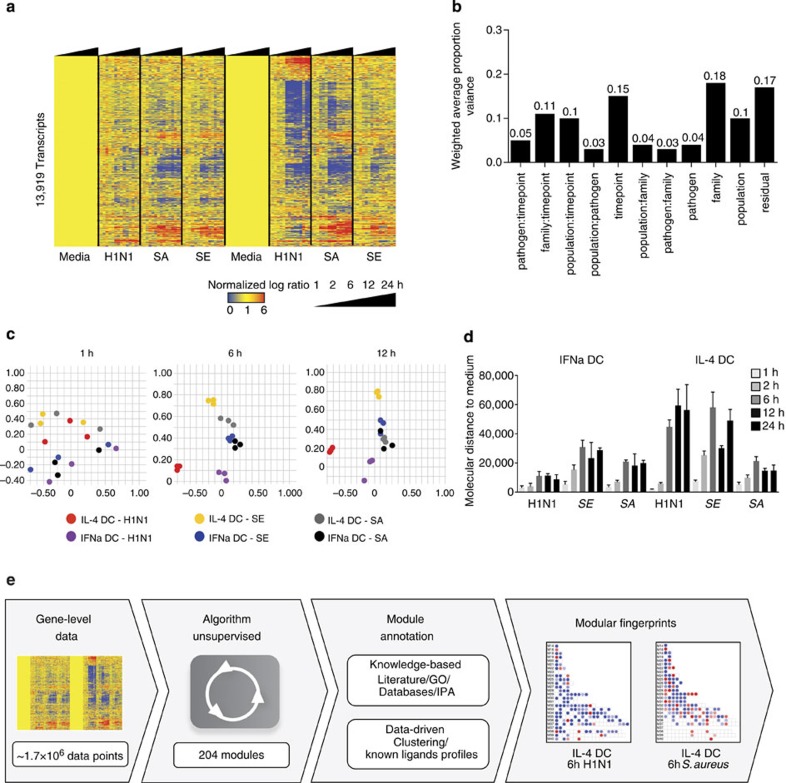
Transcriptional profiles of human DCs exposed to viral and bacterial pathogens over time. (**a**) Heatmap of the 13,919 genes differentially expressed in DCs stimulated *in vitro* with influenza H1N1, *S. enterica* (SE) or *S. aureus* (SA) for 1, 2, 6, 12 or 24 h. (**b**) PVCA of the 13,919 genes identified in **a**, including DC population, pathogen, pathogen family, time point and their interacting terms. (**c**) Principal component analysis of the 13,919 genes differentially expressed in this data set, coloured by DC population and pathogen for 1, 6 and 12 h. (**d**) Bar chart representing the mean MDTM induced by the three pathogens in IL-4 and IFNα DCs at all time points as compared with medium control. Error bars represent the s.d. (three replicates). (**e**) Workflow describing the modular framework development process. Transcript-level data from IL-4 and IFNα DC stimulated with pathogens were automatically processed for module extraction (see Methods). Modules were annotated using a combination of knowledge-based and data-driven approaches. Finally, module fingerprints were generated for each condition studied and represented as grids or heatmaps.

First, we defined the transcriptional changes elicited in DCs exposed to different pathogens over time. To this end, we generated DCs by culturing monocytes with granulocyte-macrophage colony-stimulating factor (GM-CSF) and either interleukin-4 (IL-4; hereafter IL-4 DC) or IFN-α (hereafter IFNα DC). IL-4 DCs display the most immature DC phenotype^51^, whereas IFNα DCs display a phenotype of DCs activated in viral infections or certain immune disorders such as systemic lupus erythematosus^52^. IL-4 and IFNα DCs from three healthy donors were exposed *in vitro* for 1, 2, 6, 12 and 24 h to either a virus (Influenza H1N1 Brisbane, 2007), a Gram-negative bacterium (*Salmonella enterica*) or a Gram-positive bacterium (*Staphylococcus aureus*). One-way analysis of variance yielded 13,919 transcripts differentially expressed across DCs, pathogens and time points. Figure 1a shows an overview of transcriptional changes in IL-4 and IFNα DCs in response to the three pathogens by hierarchical clustering. Principal variance component analysis (PVCA) combined with further cluster analysis demonstrated that the main contributor to transcriptional profile heterogeneity is the type of pathogen, that is, virus or bacteria (proportion variance: 0.18) followed by time point and population (proportion variances: 0.15 and 0.10; Fig. 1b, Supplementary Fig. 1). At later time points, differences between DCs in response to H1N1 could be detected. This variance between DCs in response to virus, as well as *S. enterica*, is further illustrated by principal component analysis (Fig. 1c). To quantitate these differences, we developed the molecular distance to medium (MDTM, see Methods), a numerical score of a sample’s transcriptional changes as compared to control. This approach identified significant quantitative differences between DCs’ response to pathogens at various time points (Fig. 1d). IFNα DCs showed the highest magnitude of response to *S. aureus* and *S. enterica*, while IL-4 DCs showed the highest magnitude of changes in response to H1N1 and *S. enterica*. The MDTM also highlighted differences in transcriptional kinetics, revealing that in both DCs, *S. enterica* elicited the most rapid response.

Thus, unsupervised gene-level analysis combined with quantification of signatures by MDTM reveals a broad spectrum of unique and common transcriptional responses to pathogens over time. However, the complexity of interpretation of these multi-parametric profiles necessitates novel analytical approaches.

### A modular framework to study DC transcriptomics

To facilitate the biological interpretation of this dataset, we generated an unbiased analysis framework that groups transcripts co-expressed across pathogens, time points and DCs into clusters (modules). Starting from the transcript-level data, the construction of modules proceeds from the most conserved patterns of expression across all conditions to the most specific ones (Supplementary Fig. 2). Briefly, co-expression clusters are first identified for each of the 40 stimulus groups (two cell populations, five time points, four stimuli). All pairs of transcripts considered are then assigned a score between 0 and 40 based on the number of stimulus groups in which they co-cluster and a weighted co-cluster matrix is built. Finally, a graph theory approach is used to organize groups of probes connected by the largest score into cliques, through multiple rounds of selection that go from the most conserved to the most specific clustering pattern across stimulus groups. Each clique identified forms a module, and a transcript can only appear in one module. Modules are annotated using both knowledge-based and data-driven approaches (Supplementary Table 1, see Methods). Finally, modular fingerprints are derived for all samples using their control as reference (Fig. 1e). These data-driven co-expression modules cover three major parameters including time, pathogen type and DC population.

**Figure 2:**
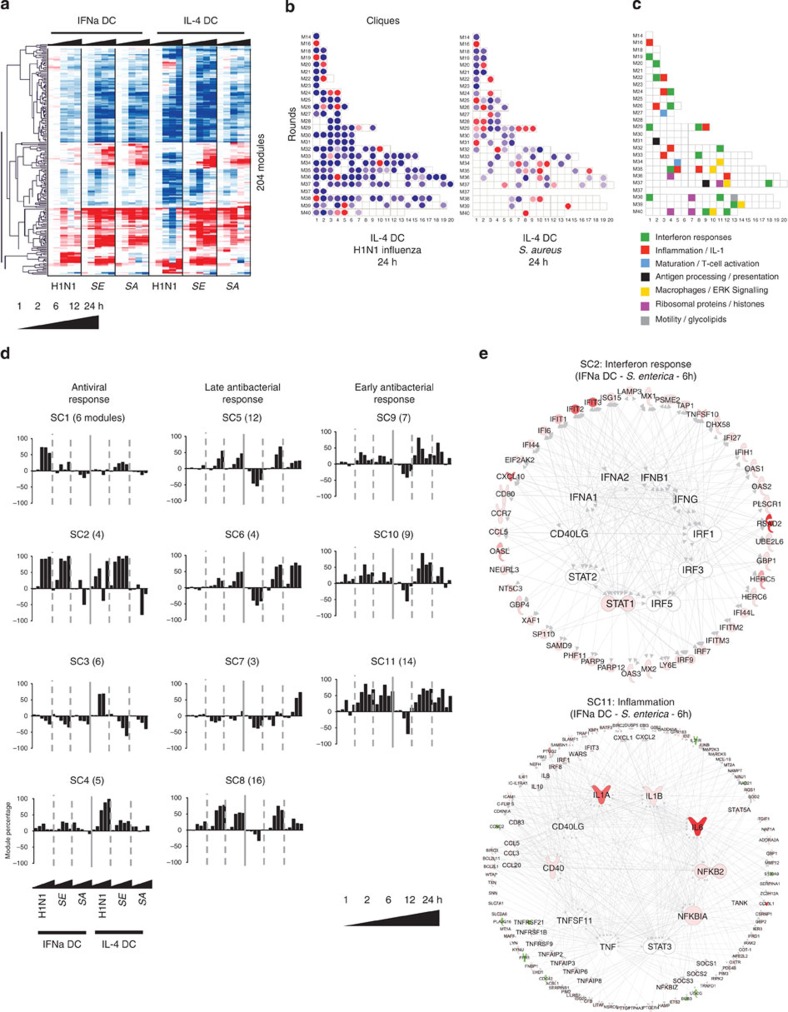
A modular transcriptional framework derived from human DCs exposed to viral and bacterial pathogens. (**a**) Hierarchical clustering (Pearson) of the 204 modules in the pathogen dataset. (**b**) Example of module grids, representing signatures induced in IL-4 DCs after 24 h challenge with H1N1 influenza or *S. aureus*. (**c**) Module functional interpretation key. (**d**) Bar charts representing the average longitudinal transcriptional profiles identified by SOTA for each cluster of modules. (**e**) IPA networks for transcripts from the module clusters C2 and C11 in IFNα DCs stimulated with *S. enterica* for 6 h. Cluster transcripts are represented on the outer circle, with representative transcripts highlighted. Predicted cytokines and transcription regulators up or downstream of SOTA clusters are represented on the inner circle. Molecules are coloured according to their fold change in the condition represented as compared with medium control.

In this fashion, 204 modules covering 6,278 transcripts altered in pathogen-stimulated DCs were identified and clustered (Pearson correlation, Fig. 2a). The scale of the module expression represents the percentage of transcripts that change twofold up (red) or down (blue) with raw data difference of at least 100 as compared with control. These fingerprints, represented as grids of modules (Fig. 2b), reduce the dimensionality of the data from 47,000 transcripts to 204 modules, facilitating the biological interpretation of altered pathways. These include the IFN response (16 modules), inflammation (10 modules), antigen processing and presentation (two modules), DC maturation and T-cell activation (two modules), motility, glycolipid metabolism, protein translation and histone regulation, extracellular signal-regulated kinase signalling and macrophage differentiation (five modules). The annotation legend of the module fingerprint is available in Fig. 2c. To facilitate public access to this framework, we developed a web application to browse the data presented in this manuscript ( http://dcmodules.com).

### Unbiased classification of modules into DC-related functions

To enable further characterization of modular expression patterns, we conducted self-organizing tree analysis (SOTA) on the 86 modules (representing 2,319 transcripts) overexpressed at least once in response to pathogens (≥40%; Supplementary Fig. 3). Eleven super clusters of modules were identified, which could be broadly divided into three categories: response to H1N1 (SOTA cluster 1 (SC1) to SC4), early response to bacteria (SC9 to SC11, with induction as early as 1 h after stimulation) and late response to bacteria (SC5 to SC8, with induction at 12 h after stimulation; Fig. 2d, Supplementary Fig. 4, Supplementary Table 2). The histogram patterns indicated that DCs responded differently to H1N1 Influenza, with IFNα DCs displaying the overexpression of a set of type-I IFN and antiviral modules (SC1), while IL-4 DCs overexpressed histones and ribosomal proteins (SC3 and SC4). In contrast, the two DC populations responded similarly to bacterial challenge, although IL-4 DCs displayed increased macrophage differentiation profile in response to *S. aureus* (SC7).

SOTA clusters were further analysed by Ingenuity Pathway Analysis (IPA) to confirm biological associations between the transcripts represented. Here transcripts from each SOTA cluster were grouped, and transcription factors and cytokines activated both upstream and downstream of these genes were identified *in silico*. The predicted regulatory pathways were represented as circular networks, with the SOTA cluster transcripts on the circumference and the transcription factors and cytokines obtained by *in silico* IPA network growth in the centre (Fig. 2e, Supplementary Fig. 5). For example, the cluster SC2 involved in antiviral responses and containing many known IFN-regulated genes, was predicted to be regulated by type-I and type-II IFNs, IFN-regulatory factors 1, 3 and 5 and STAT 1 and 2. In contrast, the cluster SC11, involved in early antibacterial responses and containing proinflammatory molecules such as NFKB2, serpinases, CD40 or TRAF1, was connected to major inflammatory molecules, including IL-1, IL-6, tumour-necrosis factor α (TNFα) and NF-κB.

Thus, SOTA clustering combined with canonical pathway analysis and *in silico* predictions helped identify groups of modules with similar behaviour in response to pathogens, improving functional annotations and regulatory understanding of modules.

### Using modules to dissect pathogen-induced signatures

**Figure 3:**
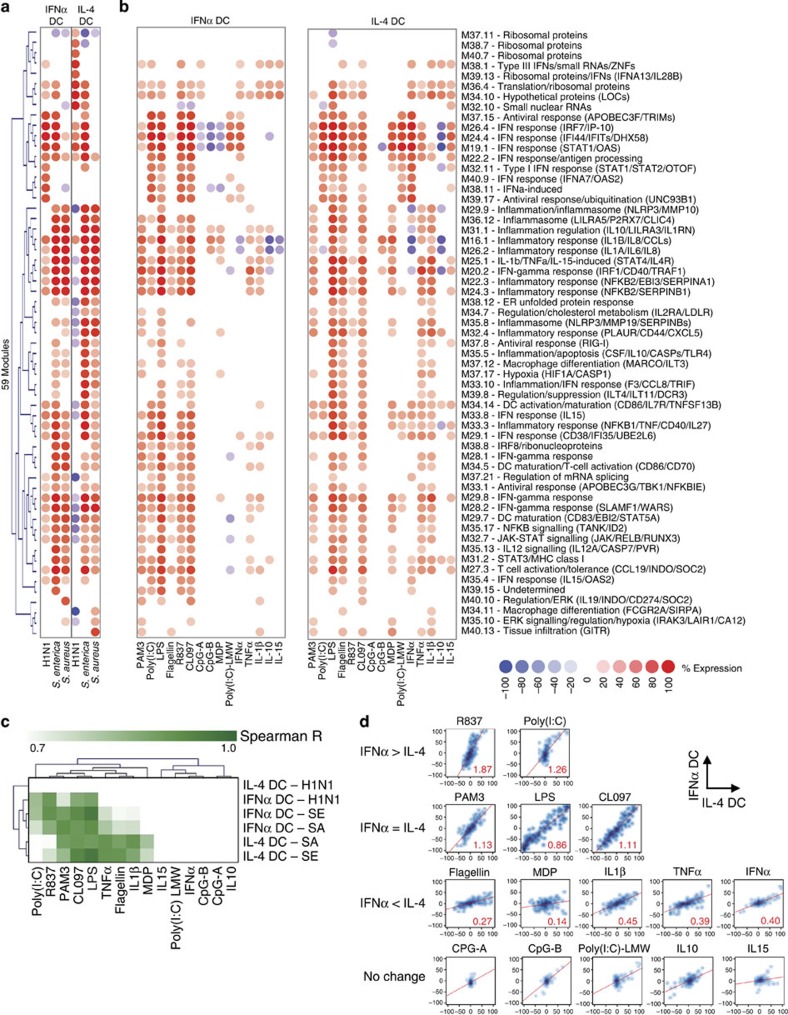
Signatures induced by pathogen components and cytokines characterize the microbial and immune regulators of module activity. (**a**) Hierarchical clustering of the 59 modules overexpressed at least once (≥40%) in DCs in response to pathogens at 6 h (average of three donors). (**b**) Induction of these 59 modules in IFNα and IL-4 DCs by microbial components and cytokines at 6 h (average of three donors). (**c**) Hierarchical clustering of Spearman *R* value from correlation analysis between profiles induced by whole pathogens and profiles induced by microbial components. (**d**) Density scatterplots representing the expression of the 204 modules in IL-4 DCs (*x* axis) or IFNα DCs (*y* axis). The red line represents the linear regression of the profiles obtained with the estimated slope in red.

We then used the annotated modules to analyze the DC transcriptional response to pathogen components and inflammatory mediators including TLR ligands, cytoplasmic receptor ligands and cytokines (Supplementary Table 3). First, we selected the 59 modules that were overexpressed at least once (≥40%) in response to any pathogen in any DC at 6 h (Fig. 3a). They were then analysed in DCs exposed for 6 h to purified ligands (Fig. 3b). Spearman correlation analysis revealed that in IFNα DC, the H1N1 signature best correlated with R837, a TLR7 agonist. The *S. enterica* signature best correlated with *Escherichia coli* lipopolysaccharide (LPS, TLR4 ligand), CL097 (TLR7/TLR8 ligand) and R837, while the *S. aureus* signature best correlated with PAM3-CSK4 (TLR2 ligand), *E. coli* LPS and CL097. In IL-4 DC, signatures from *S. enterica* and *S. aureus* correlated with TNF-α, flagellin, IL-1β and muramyl dipeptide (MDP). Interestingly, none of the microbial components and cytokines tested were able to induce the ribosomal protein modules induced by H1N1 in IL-4 DCs (Fig. 3c).

To compare the responses of different DCs, scatterplots of module expression in IL-4 (*x* axis) and IFNα DCs (*y* axis) were drawn, and the slope of the best-fit line (linear regression) was calculated (Fig. 3d). Both DCs responded similarly to PAM3 (*s*=1.13), LPS (*s*=0.86) and CL097 (*s*=1.11). However, IFNα DCs responded more to poly(I:C) (*s*=1.26) and R837 (*s*=1.87), which target TLR3 and TLR7, respectively. Conversely, IL-4 DCs responded more to molecules involved in bacterial infection, such as flagellin (*s*=0.27), MDP (*s*=0.14), TNFα (*s*=0.39) or IL-1β (*s*=0.45). This observation was supported by IL-4 DC’s increased baseline transcriptional expression of TLR5, nucleotide-binding oligomerization domain 2 and IL1R2 (Supplementary Fig. 6). As they do not express TLR9, both DCs responded poorly to CpG. Thus, different DCs are geared up to respond to distinct pathogens. This approach therefore helps unravel the biology of different DC populations.

Furthermore, this strategy can be applied to characterize the upstream microbial and immune regulators of various modules. Indeed, the modular response to IL-10 was associated with the downregulation of proinflammatory and type-I/II IFN-related modules in IL-4 DC, but the overexpression of modules containing ribosomal proteins and type-III IFNs. In contrast, recombinant IFNα downregulated proinflammatory modules, but upregulated both type-I/II and type-III IFN modules in IL-4 DCs (Fig. 3b). Thus, looking at modules elicited or repressed by purified ligands enhances our understanding of the outcome of receptor–ligand interactions.

### Specialization of APC subsets’ responses to vaccines

We then compared the responses of various APC populations with 13 commercially available vaccines, including inactivated bacterial, inactivated viral and live attenuated viral vaccines (Table 1). Circulating monocytes, IL-4 DCs, CD1c+ DCs and CD141+ DCs from four or five donors were stimulated with vaccines for 6 h, and their transcriptional fingerprints assessed by microarray. Transcripts (23,060) were detected in the combined data set incorporating the four APC populations. Hierarchical clustering revealed two groups of transcriptionally active conditions, one enriched for alum-containing vaccines and one enriched for polysaccharide-containing bacterial vaccines (Supplementary Fig. 7).

**Figure 4:**
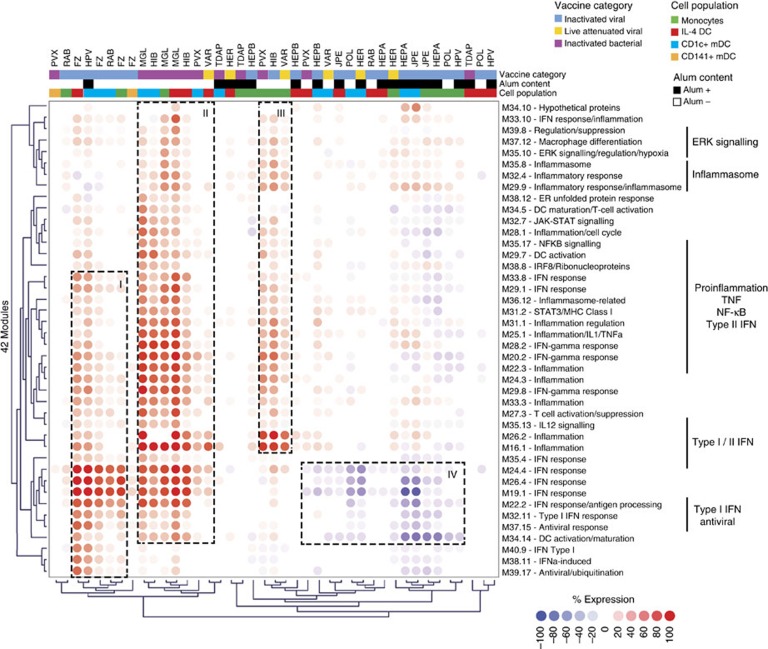
Transcriptional profiles of monocytes, IL-4 DCs, CD1c+ and CD141+ DCs stimulated with vaccines *in vitro*. Hierarchical clustering (Pearson) of the 42 modules overexpressed at least once (≥40%) in vaccine-stimulated IL-4 DCs, monocytes, CD1c+ DC or CD141+ DC. Each column represents the module expression for the four healthy donors as a group.

We then used the modules to analyze vaccine-elicited signatures. Forty-two modules were differentially expressed forming four groups (I–IV) of transcriptionally active conditions (Fig. 4). Group I included responses to viral vaccines such as Fluzone (Influenza season 09/10, FZ) and Gardasil (Human Papilloma virus, HPV), and was characterized by IFN and antiviral responses. Group II included responses to vaccines containing polysaccharides from both Gram+ and Gram− bacteria such as Menomune (*Neisseiria meningitis*, MGL), ActHib (*Haemophilus influenza*, HIB) or Pneumovax (*Pneumococcus*, PVX) in IL-4 DCs and CD1c+ DC. These signatures were characterized by the overexpression of both proinflammatory (NF-κB, inflammasome) and type-II IFN responses, with additional type-I IFN modules (M19.1, M22.2, M24.4, M26.4) detected in response to Gram− vaccines (MGL, HIB). Group III included responses to PVX, HIB and Varivax (Varicella, VAR) in monocytes only, highlighting APC-specific responses. These signatures were characterized by inflammatory (M16.1, M34.4, M35.8) and type-II IFN responses (M20.2, M29.8), with no induction of type-I IFN response modules. Finally, group IV was enriched for responses to alum-containing vaccines Havrix (Hepatitis A, HEPA), Engerix-B (Hepatitis B, HEPB) and Ixiaro (Japanese encephalitis, JPE). These signatures were present in monocytes, IL-4 DCs and CD1c+ DC, and were characterized by modest upregulation of the inflammasome (M29.9), macrophage differentiation (M37.12) and downregulation of both DC maturation (M34.5, M34.14) and the IFN response.

**Figure 5:**
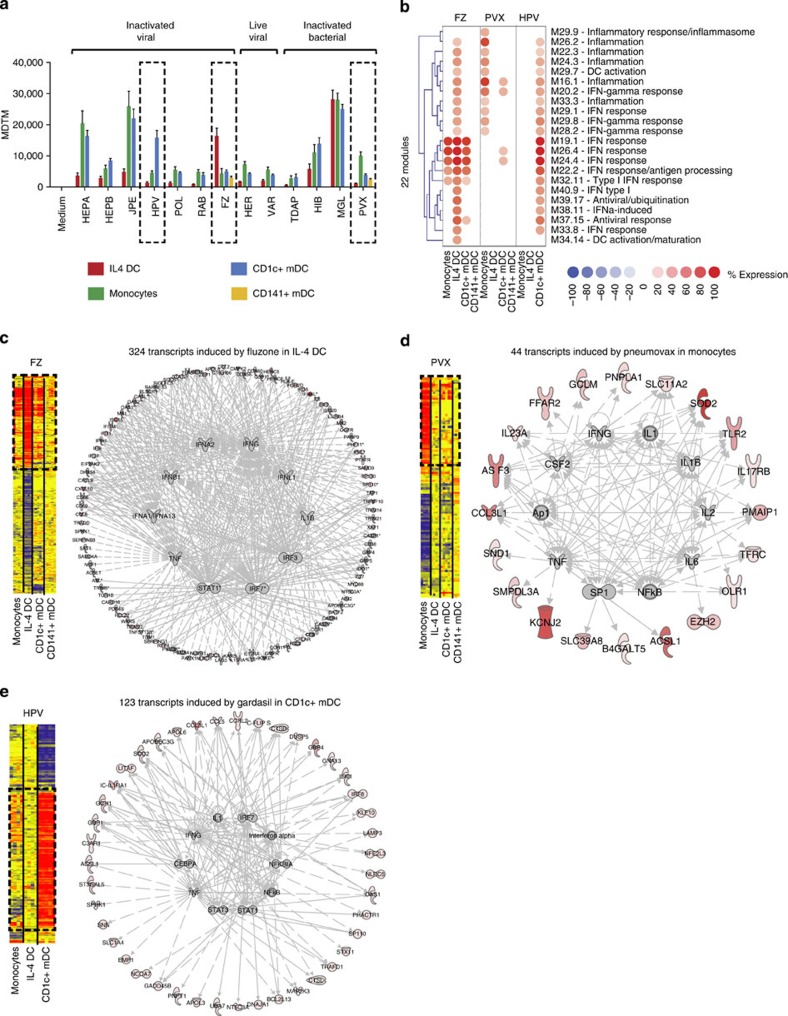
Population-specific transcriptional changes in response to PVX, FZ and HPV vaccines. (**a**) Bar chart representing the MDTM derived from the 23,060 transcripts detected in monocytes, IL-4 DCs or blood mDCs stimulated with vaccines *in vitro*. Error bars represent the s.d. (**b**) Selection of the 22 modules overexpressed 40% in at least one of the four populations in response to PVX, FZ or HPV. (**c**) Network analysis of transcripts induced by Fluzone. Left panel: hierarchical clustering of transcripts differentially expressed by Fluzone. Right panel: IPA regulation networks of transcripts overexpressed by Fluzone in IL-4 DC. (**d**) Same as **c** for transcripts overexpressed by Pneumovax in monocytes. (**e**) Same as **c** for transcripts overexpressed by Gardasil in CD1c+ DCs.

MDTM analysis further showed that HEPA, JPE, HPV, FZ, HIB, MGL and PVX vaccines elicited significant transcriptional changes in at least one of the four APC populations considered (Fig. 5a). The response to MGL vaccine, presumably driven by LPS, was similar between monocytes, IL-4 DCs and CD1c+ DC. The alum-containing vaccines HEPA and JPE induced high MDTM in monocytes and CD1c+ DCs but not in IL-4 DCs. Interestingly, three vaccines displayed population-specific transcriptional changes. HPV induced a high MDTM in CD1c+ DCs only, while FZ and PVX induced a high MDTM in IL-4 DCs and monocytes, respectively. While we could only test two vaccines in CD141+ DCs due to the low numbers that could be isolated from blood, it is noticeable that both PVX and FZ elicited very restricted transcriptional responses in these cells.

To further analyze these responses, the 22 modules induced by FZ, PVX or HPV in IL-4 DCs, monocytes or CD1c+ DCs were selected. While FZ induced four IFN response modules in monocytes, IL-4 DCs and CD1c+ DCs, it additionally induced proinflammatory, antiviral and type-II IFN response modules in IL-4 DCs only. PVX induced modular signatures in monocytes only, including IL-1-, NF-κB- and type-II IFN-related modules. Finally, HPV induced type-I and type-II IFN responses as well as proinflammatory modules in CD1c+ DCs only (Fig. 5b). The specific signatures observed in these three populations were highly reproducible between donors (Supplementary Fig. 8) and confirmed at the transcript level through regulatory pathway analysis (Fig. 5c–e).

Thus, different vaccines activate different APC populations.

### Vaccines induce various DC maturation profiles

**Figure 6:**
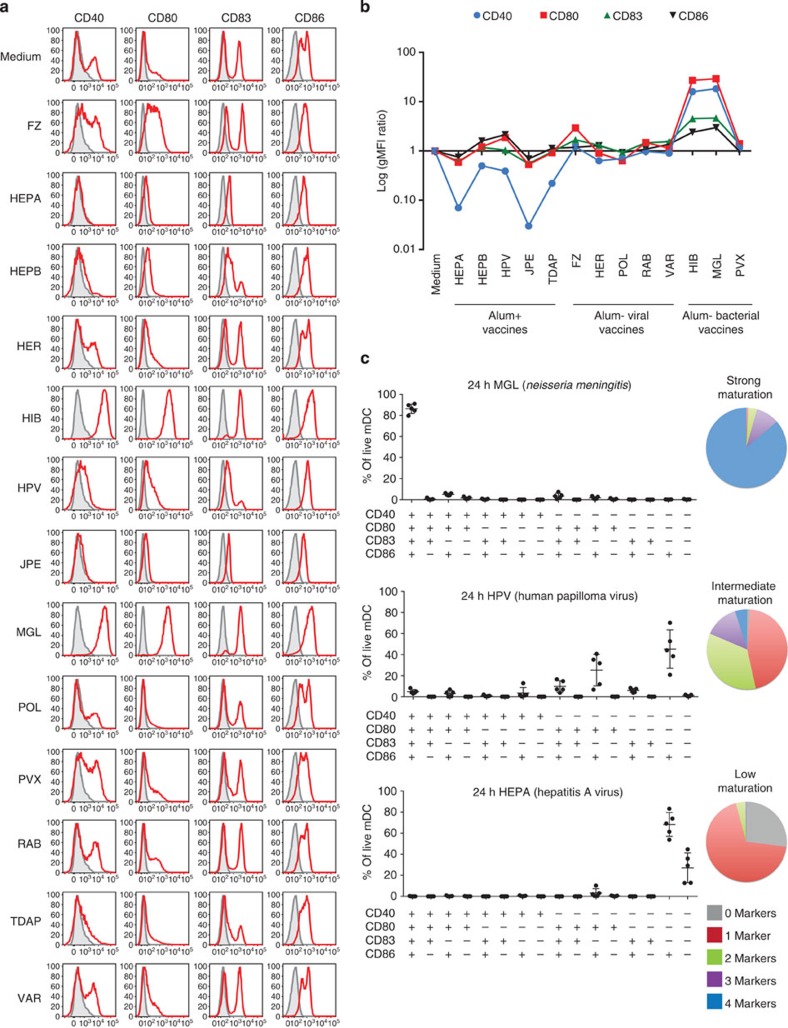
Vaccines display a broad range of CD1c+ DCs maturation capacity. (**a**) Histograms representing the expression of CD40, CD80, CD83 or CD86 on the surface of CD1c+ DCs in response to vaccine stimulation. The grey area represents the marker’s baseline expression after sort. (**b**) Line chart representing the ratio of geometric mean fluorescence intensity for CD40, CD80, CD83 and CD86 in response to each vaccine as compared with medium control for live CD1c+ DCs, measured by flow cytometry. (**c**) Boolean gating analysis of CD40, CD80, CD83 and CD86 expression for three vaccines representative of different DC maturation levels. The proportion of live CD1c+ DC expressing 0, 1, 2, 3 or 4 markers is represented as a pie chart on the right. Error bars represent the s.d.

To complement transcriptional analyses, we assessed the capacity of vaccines to induce maturation of CD1c+ DCs after 24 h stimulation. To this end, we measured the surface expression of the hallmark DC maturation and T-cell co-stimulation markers HLA-DR, CD40, CD80, CD83 and CD86 by flow cytometry. After 24 h in culture, most DCs upregulated CD86, regardless of activation conditions (Fig. 6a). The LPS-containing MGL and HIB were the most potent at increasing the expression of these activation markers on the surface of CD1c+ DCs, while HEPA and JPE, two alum-adjuvanted vaccines, decreased their expression when compared with medium control, in particular that of CD40 (Fig. 6b, Supplementary Fig. 9). Differences between combinations of markers induced were also observed by Boolean gating. While PVX and FZ induced CD40, CD83 and CD86 expression to comparable levels, FZ induced more CD80+ DCs. Boolean gating further revealed the DC maturation capability of each vaccine. MGL induced the expression of all maturation markers on CD1c+ DCs, while HPV led to intermediate levels of DC maturation, inducing only CD80 and CD86. Finally, HEPA led to low levels of DC maturation, with the majority of cells triple negative for CD40, CD80 and CD83 (Fig. 6c, Supplementary Fig. 10). The induction of these maturation markers was also confirmed at the transcriptional level at 6 h (Supplementary Fig. 11a), and highly correlated with their geometric mean fluorescence intensity protein change ratio at 24 h (Supplementary Fig. 11b).

Altogether, these observations highlight significant differences between vaccines in their capacity to directly activate and induce maturation of DCs.

### Vaccine signatures in individuals exposed to influenza

**Figure 7:**
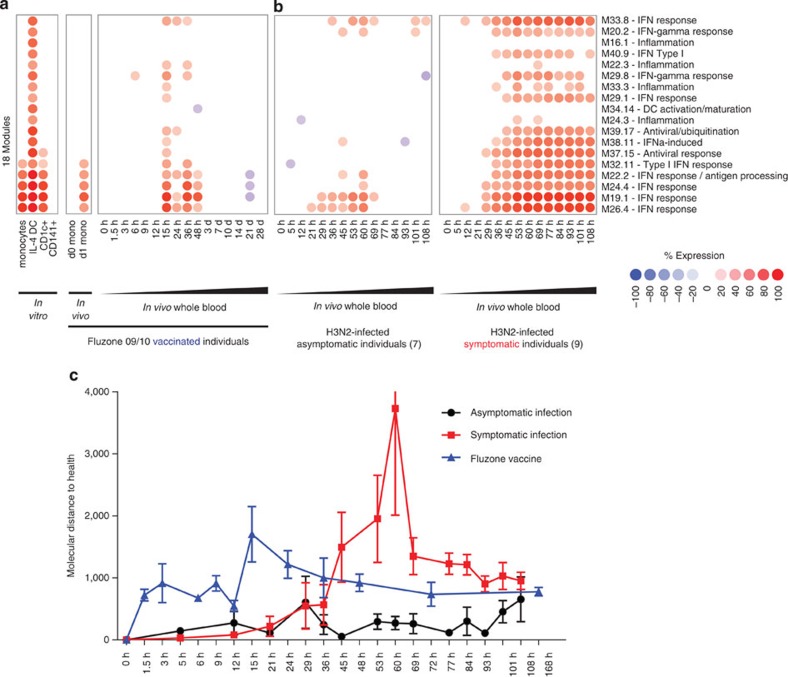
Transcriptional signatures induced by fluzone in APC *in vitro* are detectable *in vivo* in the blood of fluzone-vaccinated individuals and H3N2-infected individuals. (**a**) Module fingerprints induced *in vitro* by fluzone in isolated monocytes or whole blood of healthy subjects vaccinated with fluzone. (**b**) Fingerprints from the modules from **a** in asymptomatic (average of seven donors, left panel) and symptomatic (average of nine donors, right panel) H3N2-infected individuals monitored over 108 h. (**c**) Molecular distance to health analysis for both fluzone vaccination and H3N2 infection cohorts. The 0-h time point is used as reference for each individual.

We applied our *in vitro*-derived module framework to analyze the blood transcriptional profiles of subjects exposed to flu vaccines and influenza. Modular fingerprints were generated from blood transcriptional profiles of 15 healthy individuals from three cohorts vaccinated with Fluzone, obtained at 16 time points after vaccination (0, 1.5, 3, 6, 9, 12, 15, 24, 36, 48 h, 3, 7, 10, 14, 21 and 28 days)^40^. The five IFN modules induced *in vitro* by FZ in monocytes, IL-4 DCs and CD1c+ DCs were detected *in vivo* in monocytes isolated 24 h after vaccination. In whole blood from vaccinated individuals, these modules were overexpressed as early as 15 h after vaccination, and returned to baseline expression by day 3 (Fig. 7a). The dynamics of this signature were supported by the quantitative changes measured by molecular distance to health analysis (Fig. 7c). This suggests that vaccination with Fluzone elicits temporary transcriptional changes in the circulating myeloid compartment.

To determine the expression of DC modules during flu infection, we generated modular fingerprints from publicly available blood transcriptional profiles of nine symptomatic and seven asymptomatic individuals inoculated with live influenza (H3N2/Wisconsin) and monitored at 15 different time points over 108 h (ref. 34). Of note, our Illumina-based modular framework enabled the analysis of signatures generated with Affymetrix arrays, highlighting cross-platform usability. Asymptomatic individuals displayed a mild signature similar to that of individuals vaccinated with Fluzone, with the overexpression of three IFN response modules between 29 and 60 h post inoculation (Fig. 7b, left panel). Conversely, symptomatic individuals displayed much stronger modular response, with most modules overexpressed by 36 h and sustained up until 108 h post inoculation, including modules linked to IFNα and antiviral responses (Fig. 7b, right panel). The intensity and sustainability of this response was confirmed by molecular distance to health analysis (Fig. 7c). Interestingly, at 15 h, vaccinated individuals displayed the upregulation of modules linked to NF-κB-driven inflammation (M22.3), IFN-γ response (M29.8), TNF and CD40 signalling (M33.3). These pathways, involved in T-cell activation and development of adaptive immunity, were detected in symptomatic individuals, but not asymptomatic ones.

These observations show that our APC module-based approach permits the dissection of transcriptional responses in whole blood *in vivo,* highlighting commonalities and differences between immune responses to asymptomatic and symptomatic flu infections and flu vaccination.

## Discussion

The rapidly progressing field of systems biology has enabled the identification of predictors of vaccine immunogenicity and is offering insights into the mechanisms of action of vaccines^38^,^50^,^53^,^54^. Furthermore, comparing systemic immune responses elicited by different vaccines has enhanced our understanding of the immune mechanisms underpinning successful vaccination^40^,^50^. However, vaccines against a number of infectious agents are still not effective and even successful vaccines are not effective in all individuals. This, for example, is well documented in the elderly^46^. Thus, the rational design of next-generation vaccines, including antigen choice, delivery method and adjuvant selection, would benefit from better understanding how vaccines interact with both the innate and adaptive arms of the immune system.

Herein, we provide a systems biology-based characterization of human APC transcriptional responses to vaccines. To interpret the signatures obtained, we generated a modular framework based on the transcriptional responses of DCs to pathogens over time. The modules were further dissected in a data-driven fashion by identifying known microbial components and human cytokines affecting their expression. We applied this framework to study the early transcriptional responses of IL-4 DCs, CD1c+ DCs, CD141+ DCs and monocytes to 13 vaccines *in vitro*. We observed polarization of CD1c+ DC response to Gardasil (HPV), IL-4 DCs response to Fluzone (influenza) and monocyte response to Pneumovax (*pneumococcus*), suggesting that responses to different vaccines may be mediated through unique APC subsets. Finally, we applied these modules to analyze blood transcriptional profiles from individuals vaccinated with Fluzone and individuals with symptomatic and asymptomatic H3N2 infections. To facilitate further dissemination and cooperative analysis of the data presented herein, we developed an interactive web application available at http://dcmodules.com.

We detected strong modular signatures in blood transcriptional profiles from individuals vaccinated with Fluzone, supporting that this approach can complement our previously described whole-blood modular analysis^40^ by providing increased resolution of altered myeloid signals in distinct immunological states. We could also derive DC module fingerprints from blood profiles of individuals infected with H3N2, which were generated using a different microarray platform. While we found the most transcriptional similarities between vaccinated individuals and individuals with asymtomatic H3N2 infections, vaccinated individuals shared adaptive immune system activation profiles with infected symptomatic individuals. This approach may thus enable the segregation of immune pathways required for vaccination from those that drive pathogenesis during infection. Identifying the ‘good’ pathways from the ‘bad’ should help in the development of more efficacious and safer vaccines.

We recently reported the early induction of the IFN response by Fluzone in the blood of vaccinated subjects^40^. The IFN signature of monocytes isolated from the blood of individuals vaccinated with Fluzone is remarkably similar to that of Fluzone-stimulated monocytes *in vitro*, suggesting that this population may play an important role in the process of vaccination. Interestingly, inflammatory monocytes suppressed vaccine immunity in a mouse model of ovalbumin and hemagglutinin vaccination^55^. Whether these transcriptionally activated monocytes affect the outcome of Fluzone vaccination in human remains to be established.

In this study, vaccines induced a wide range of maturation profiles in CD1c+ DC. MGL and HIB vaccines were the most potent at inducing mDC maturation, corroborating recent studies by Li *et al*.^50^ These two vaccines contain detectable levels of LPS (data not show), which is known to induce DC maturation through a TLR4-dependent mechanism. FZ and PVX also induced DC maturation, albeit to a lower extent. Noticeably, alum-adjuvanted vaccines including HEPA, HEPB, JPE, TDAP and HPV were poor inducers of DC maturation. Transcriptionally, most of these vaccines weakly activated the inflammasome^56^,^57^, as well as lipid metabolism and hypoxia programs. Interestingly, they downregulated a DC maturation module containing CD86 and MHC Class I molecules, which are essential for antigen presentation to CD8^+^ T cells. This suggests that alum does not directly activate DCs, but conducts its immunogenic activity through the recruitment of alternate inflammatory cell populations. Interestingly, HPV was the only alum-adjuvanted vaccine that could elicit both IFN and proinflammatory response in CD1c+ DCs at 6 h, and the one that could induce CD80+ and CD83+ cells. This suggests that the HPV vaccine contains other immunogenic components, detected only by CD1c+ DCs. Altogether, these observations bring into perspective the selection of adjuvant during vaccine design, which in addition to global immunogenicity and activation of APC, should consider the specific type of immune responses induced in APC. In this context, the development of vaccines specifically targeted to DCs and combined with adjuvants such as specific TLR ligands may improve long-term protective immunity against pathogens.

The DC subset-specific transcriptional profiles obtained in response to FZ, PVX and HPV are presumably driven by differences in PRRs expressed in each cell type. Using APC populations with well-defined phenotypes may help us further decorticate the effect of each vaccine component on immune cells. For example, Langerin, which is transcriptionally overexpressed in CD1c+ DCs as compared with monocytes or IL-4 DCs (Supplementary Fig. 12), is a known receptor for the HPV protein HPV16, present in Gardasil. This interaction may be involved in the onset of the inflammatory cascade observed at 6 h. Monocytes express a variety of lectins that detect large polysaccharides, which may explain why they respond more to PVX stimulation. Our data demonstrate that IL-4 DCs respond differently than monocytes or circulating DCs to vaccines. While monocyte-derived DCs remain a useful and amenable *in vitro* model to define pathogen-induced molecular changes, our results highlight the importance of considering the specific functions of DCs circulating *in vivo*. Similarly, we can presume that skin-resident DCs such as Langerhans cells and dermal DCs, which may first detect vaccine antigen, will display distinct response patterns to vaccines, and should therefore be included in future studies.

Systems biology approaches generate high-dimensional data sets that require trained analysts and computational tools to extract biologically relevant information. A major goal behind the development of co-expression frameworks is the reduction of data dimensionality^46^. Such tools should provide investigators with accelerated paths to biological discovery, through intuitive data computation and visualization. Herein, we let the data generated from DCs stimulated with pathogens, microbial components and human cytokines guide the development and interpretation of the identified modules. These modules recapitulate some of the transcriptional programmes previously described in APC^41^,^58^, including the IFN response, the NFκB-driven inflammation or the inflammasome, which serves as a validation of this approach. In addition, the modules permit further stratification of these major innate immunity networks. For example, the modules can distinguish between type-I, -II and -III IFN responses, which show distinct induction profiles in response to different pathogens. We were intrigued by the type-II IFN modules, since the production of IFNγ by human DCs remains a subject of debate. It is likely that these modules can also be induced by other inflammatory mediators, such as TNFα or IL-1β (Fig. 3), highlighting the redundancy of innate signalling networks and the challenges in functional annotations.

Finally, the unbiased grouping of transcripts according to similar expression patterns across conditions permits the expansion of known networks and the formulation of testable hypotheses about the role of new molecules in these pathways. Knowledge-based network connectivity analyses permit the identification of major known regulators (cytokines and transcription factors) of these modules, as shown in Fig. 2e, highlighting molecular hubs controlling the activation/repression of DC function. As expected, IFNAs, IFNB1, IRF1-3-5 and STAT1-2 were connected with the main group of IFN modules (SC2), while IL1B, IL-6, NFKB2, STAT3 and TNF were connected to proinflammatory modules (SC11). It will be interesting to look at the connectivity within other module groups, including SC3 and SC4, which combine ribosomal proteins, histones and type-III IFNs for example (Supplementary Fig. 5). Future studies should assess how the interruption of major transcription regulators identified *in silico* affects each module, and how vaccines can be designed to activate/repress these molecules.

Overall, the characterization of DC transcriptional responses to current vaccines improves our understanding of their mechanisms of action, by identifying the activation signals they elicit in specific APC populations. Vaccine components such as antigens and adjuvants can be further tested to determine APC maturation ability and the type of adaptive immunity subsequently induced. This will yield essential information for the development of the next-generation preventive and therapeutic vaccines, where targeting of specific APC populations through surface receptors will permit the induction of highly specific cellular and/or humoral adaptive immune responses.

## Methods

### Ethical statement

All protocols were reviewed and approved by the Institutional Review Board (IRB 012-089) at Baylor Research Institute (Dallas, TX). Written informed consent was obtained from all healthy donors.

### Monocyte-derived dendritic cell cultures

Monocytes were obtained from frozen fraction 5 from healthy donor apheresis. Cells were thawed and resuspended in PBS. Cells were spun at 350 *g* for 7 min, washed with 1 × PBS, counted, washed again and resuspended in PBS/2% fetal bovine serum/1 mM EDTA at 5 × 10^7^ cells ml^−1^. Monocytes were enriched using the EasySep Human Monocyte Enrichment Without CD16 Depletion Kit (StemCell Technologies) according to the manufacturer’s protocol. Once enriched, cells were resuspended in serum-free CellGenix DC medium (CellGenix, Germany)/1% penicillin/streptomycin at 10^6^ cells ml^−1^. GM-CSF (Leukine, Genzyme Corporation) was added at 200 ng ml^−1^. For IL-4 DC, recombinant IL-4 was added at 50 ng ml^−1^ and cells were fed a full dose of GM-CSF and IL-4 at day 2 and day 4 of a 6-day culture. For IFNα DC, IFNα was added at 500 U ml^−1^, and cells were fed a full dose of GM-CSF and IFNα at day 1 of a 3-day culture. Cell suspensions were injected into 72 ml sterile culture bags (AFC, Gaithersburg, MD). Cells were cultured at 37 °C in 5% CO_2_ atmosphere.

### CD1c+ and CD141+ DC isolation

Human blood DCs were isolated from PBMCs using leukapheresis products. DCs were first enriched using human Pan-DC Pre-Enrichment Kit (StemCell Technologies) and then stained with fluorochrome-conjugated specific antibodies including Lineage cocktail 1-FITC (BD, dilution: 1:10), CD11c-V450 (B-ly6, BD, dilution: 1:50), CD1c/BDCA1-PerCp-Cy5.5 (L161, Biolegend, dilution 1:25), HLA-DR-APC-eFlour780 (LN3, eBioscience, dilution: 1:50), CD303/BDCA-2-PE (AC144, Mitenyi Biotec, dilution: 1:25) and CD141/BDCA3-APC (AD5-14H12, Mitenyi Biotec, dilution: 1:25). After washing with PBS, DCs were sorted as Lineage-HLA-DR+CD11c+ cells differentially expressing CD1c and CD141 using FACSAria II with Diva software (BD).

### Monocyte-derived dendritic cell stimulation

After culture, cells were collected, washed in PBS and resuspended in complete RPMI (1 l RPMI, 10 ml penicillin/streptomycin, 10 ml L-glutamine, 10 ml non-essential amino acids, 10 ml sodium pyruvate, 25 ml HEPES, 500 μl β-mercaptoethanol 1,000 × ) at 10^6^ cells ml^−1^. Stimulations were conducted in 96-well 1-ml deep-well plates (Greiner Bio-One) in 500 μl (5 × 10^5^ cells). All stimuli used are summarized in Supplementary Table 3. At the end of stimulation, cells were spun at 350*g* for 7 min, washed with PBS, spun again and lysed in 600 μl (for 1 × 10^6^ cells) or 350 μl (for 5 × 10^5^ cells) of RLT buffer, Qiagen). Cell lysates were stored at −80 °C until extraction.

### Influenza virus propagation

Madin Darby Canine Kidney cells were grown to 80% confluence in 75 cm^2^ flask. The cells were infected with A/Brisbane/59/2007 IVR 148 (H1N1) strain at a ratio of 1:100 for 1 h. The cells were washed twice and incubated for 48–72 h with media (Opti-MEM, Life Technologies). Supernatant was harvested post infection. The virus titre from the supernatant was assessed by plaque assay. Experiments were performed under BSL-3 laboratory conditions.

### *Salmonella enterica* culture

Bacteria were thawed and plated on a nutrient agar (Difco) plate with a sterile 10 μl inoculating loop, incubated overnight at 37 °C and subsequently stored at 4 °C. A single colony was picked up from the agar plate and inoculated in 6 ml Difco nutrient broth (in 15 ml Falcon tube), and cultured overnight at 37 °C on a shaker. After expansion, bacteria were brought in log phase by culturing 500 μl of bacterial preparation in 4.5 ml fresh broth for 1.5 h at 37 °C on a shaker. Bacteria were heat killed for 30 min in 72 °C water bath and stored at −80 °C until use.

### Monocyte-derived dendritic cell stimulation with pathogens

IL-4 and IFNα DCs were stimulated *in vitro* for 1, 2, 6, 12 or 24 h with either live influenza virus H1N1 Brisbane (multiplicity of infection 5:1), heat-killed *Salmonella enterica* (10^8^ c.f.u. ml^−1^, ATCC# 14028) or heat-killed *Staphylococcus aureus* (10^8^ c.f.u. ml^−1^, Invivogen) at 37 °C in 5% CO_2_ atmosphere. All pathogen stimulations were conducted in 96 deep-well plates as described above. At the end of incubation time, cells were washed twice with PBS, lysed in the plate 350 μl RLT buffer (RNeasy Kit, Qiagen) and stored at −80 °C.

### mRNA preparation and hybridization

Total RNA was isolated from cell lysates using the RNeasy Mini-Kit (Qiagen) according to the manufacturer’s instructions. Following extraction, an Agilent 2100 Bioanalyzer (Agilent, Palo Alto, CA) was used to measure RNA Integrity Numbers for each sample. All samples with RNA Integrity Number values greater than 7 were retained for further processing. RNA concentration was measured using a Nanodrop 1000 (Nanodrop Technologies, Wilmington, DE). RNA (250 ng) from all samples passing quality control were amplified and labelled using the Illumina TotalPrep-96 RNA amplification kit (Ambion, Austin, TX). Amplified labelled RNA (750 ng) were hybridized overnight to Illumina HT12 V3 or V4 beadchips (Illumina, San Diego, CA). Chips were scanned on an Illumina BeadStation 500 following the manufacturer’s protocols.

### PVCA

The weighted average proportion variance was calculated with the R/Bioconductor package ‘pvca’^59^ following the author’s instructions. The factors ‘cell population’, ‘pathogen’, ‘pathogen family’ and ‘time point’ were considered. The threshold used for the minimum amount of the variabilities explained by the selected principal components was 0.5. The PVCA was run on the normalized data, using the 13,919 transcripts differentially expressed in DCs stimulated with pathogens.

### MDTM/health

The MDTM is calculated for all samples as the sum of absolute fold-changes ≥2 as compared with the samples’ medium reference control for an arbitrary gene list. Herein, we chose the list of genes present at least once according to Illumina detection *P* value (*P*<0.01 in at least one sample), and over or underexpressed twofold at least once in the data set analysed. The molecular distance to health is the *in vivo* counterpart of the MDTM, where the reference controls come from healthy individuals.

### Module construction algorithm and fingerprints

The module construction process is summarized in Supplementary Fig. 2. To derive modular fingerprints, the expression of each module is calculated independently. For each gene belonging to a module, we assess whether that gene is overexpressed (≥ twofold up, 100 raw data difference as compared with reference sample), underexpressed (≥ twofold down, 100 raw data difference) or unchanged. For *ex vivo* whole blood, monocytes and neutrophil modular signatures, we used 1.2-fold threshold, as the global intensity of transcription profiles is dimmer. The percent of transcripts overexpressed and underexpressed are calculated and subtracted from each other. For each sample, a percent expression score is assigned to each module (between −100 and 100%) and represented on a grid. The module expression is represented as coloured circles (red: overexpression, blue: underexpression) and the intensity of the color represents the percentage (bright ~100% to dim ~0%). The modular fingerprints are represented on two-dimensional grids with module extraction rounds as rows, and modules extracted per round as columns. Module 14.1 is the cell on row 14, column 1 (Fig. 2b).

### Module annotations

To better interpret fingerprints, modules were associated with known functions or molecular pathways. We first annotated modules according to their enrichment in transcript linked to the IFN response (IFNs and IFN-inducible transcripts) or the inflammatory response (NF-κB-inducible transcripts, inflammasome, TNF signalling)^41^. Modules were further annotated using gene ontology enrichment, IPA, Genemania and BioGPS. A legend is available on Fig. 2c.

To provide an unsupervised data-driven functional interpretation of modules, we classified them according to their level of expression following DC activation with known pathogen-associated molecular patterns and cytokines (Supplementary Table 3).

### Vaccines and working vaccine concentrations

We selected 13 commercially available vaccines that are delivered in soluble form intradermally or intramuscularly. The vaccines used are summarized in Table 1, including manufacturer, alum content and working concentration. To assess vaccine activation concentration, we cultured IL-4 DCs with three different concentrations of each vaccine for 24 h and assessed DC viability and activation status by flow cytometry. DC and monocyte purity were assessed with the following panel: FITC-CD16, PerCP-Cy5.5-CD14, PE-CD141, ECD-CD19, PE-Cy7-CD56, APC-CD1c, Alexa Fluor 700-HLA-DR, APC-H7-CD8 and V450-CD3. The purity analysis gating strategy is summarized in Supplementary Fig. 13a. Viability was assessed by the incorporation of Live/Dead Fixable Yellow dye (Life Technologies; Supplementary Fig. 13b,c). DC activation/maturation was assessed with the following panel: FITC-CD56, PerCP-Cy5.5-CD14, PE-CD1a/b/c, ECD-CD16, PE-Cy7-CD40, APC-CD83, Alexa Fluor 700-CD3/CD19/CD20, APC-H7-CD80, V450-CD86 (Supplementary Fig. 14). We selected the stimulation concentration that best activated IL-4 DCs while retaining elevated viability, and used these concentrations for CD1c+ DC maturation experiments. The stimulation concentrations are summarized in Table 1.

### Web interface and GSEA geneset

All module data presented in this manuscript can be browsed online at http://dcmodules.com. The application was developed in Ruby, using the Ruby on Rails web development framework, with Postgresql database support, and is hosted in the cloud. The application lists all samples generated and analysed in this manuscript. It lists the 204 modules composing the analytical framework described, with annotations and transcript composition. It provides multiple ways for the user to visualize data, including individual module maps, multi-sample module heat maps, line charts and scatterplots. In addition, it allows the user to filter the module expression data based on module expression across experiments or subsets of samples. In addition, we provide the module framework as a. gmx file (Supplementary Material) for Gene Set Enrichment Analysis.

## Additional information

**Accession codes:** The data sets described in this manuscript are deposited in the NCBI Gene Expression Omnibus (GEO, http://www.ncbi.nlm.nih.gov/geo, GEO Series accession numbers GSE44719, GSE44720, GSE44721, GSE44722, GSE56744). Data sets for *in vivo* vaccination and H3N2/Wisconsin infection analyses were previously published^34^,^40^ and are available in GEO (GSE30101, GSE30550). Additional sample, experiment and module information as well as interactive data displays are available through the web interface described above at http://dcmodules.com.

**How to cite this article**: Banchereau, R. *et al.* Transcriptional specialization of human dendritic cell subsets in response to microbial vaccines. *Nat. Commun.* 5:5283 doi: 10.1038/ncomms6283 (2014).

## Supplementary Material

Supplementary Information Supplementary Figures 1-14, Supplementary Tables 1-3.

Supplementary Data 1 [Gene Set Modules] Supplementary Data 1

## Figures and Tables

**Table 1:** Vaccine list.

**Pathogen**	**Vaccine**	**Abbreviation**	**Type**	**Alum (mg** **ml^−1^)**	**Stimulation concentration (μl** **ml^−1^)**
*Haemophilus influenza*	ActHib (Sanofi)	HIB	Inactivated bacterial	0	30
Hepatitis A	Havrix (GSK)	HEPA	Inactivated viral	0.5	60
Hepatitis B	Engerix-B (GSK)	HEPB	Inactivated viral	0.5	60
Herpes Zoster (Shingles)	Zostavax (Merck)	HER	Live attenuated viral	0	30
Human Papilloma virus	Gardasil (Merck)	HPV	Inactivated viral	0.45	30
Influenza	Fluzone 09-10 (Sanofi)	FZ	Inactivated viral	0	6
Japanese encephalitis	Ixiaro (Novartis)	JPE	Inactivated viral	0.5	60
*Neisseiria meningitis*	Menomune (Sanofi)	MGL	Inactivated bacterial	0	60
Pneumococcus	Pneumovax (Merck)	PVX	Inactivated bacterial	0	60
Polio virus	Ipol (Sanofi)	POL	Inactivated viral	0	60
Rabies	Imovax (Sanofi)	RAB	Inactivated viral	0	60
Tetanus, Diphteria, Pertussis	Adacel (Sanofi)	TDAP	Inactivated bacterial	0.66	6
Varicella	Varivax (Merck)	VAR	Live attenuated viral	0	60
