# Supplementary material for: Transcriptional specialization of human dendritic cell subsets in response to microbial vaccines
Source: Nat Commun. 2014 Oct 22;5:5283. doi: 10.1038/ncomms6283 (PMC4206838; doi:10.1038/ncomms6283)
Supplement: Supplementary Information — Supplementary Figures 1-14, Supplementary Tables 1-3. [file ncomms6283-s1.pdf]

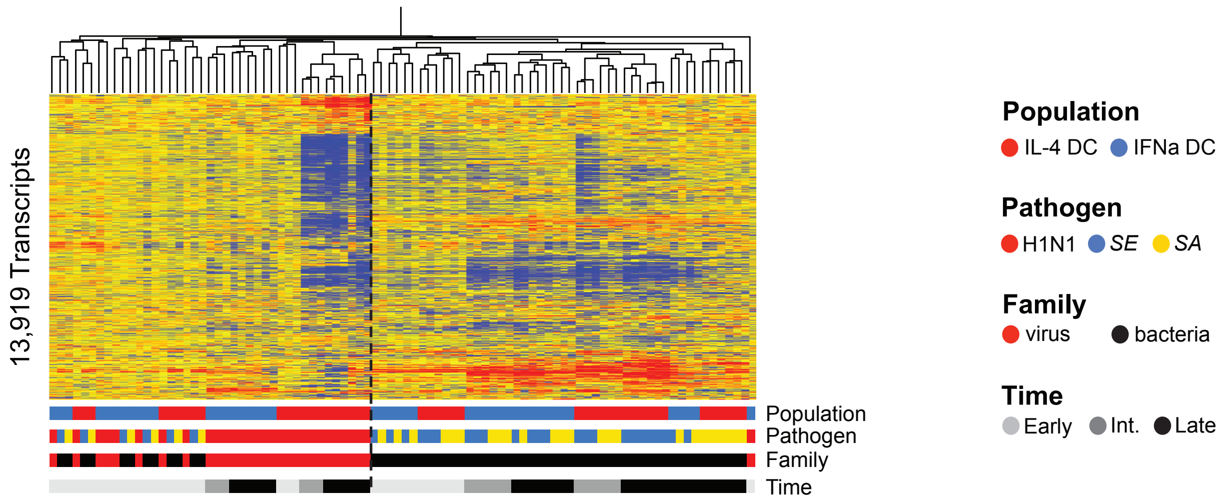

Supplementary Figure 1 - Hierarchical clustering of samples according to the expression of the 13,919 transcripts modulated in IFNα and IL-4 DC stimulated with pathogens, colored by DC population, pathogen, pathogen family or time (early: 1h, 2h; intermediate: 6h; late: 12h, 24h).

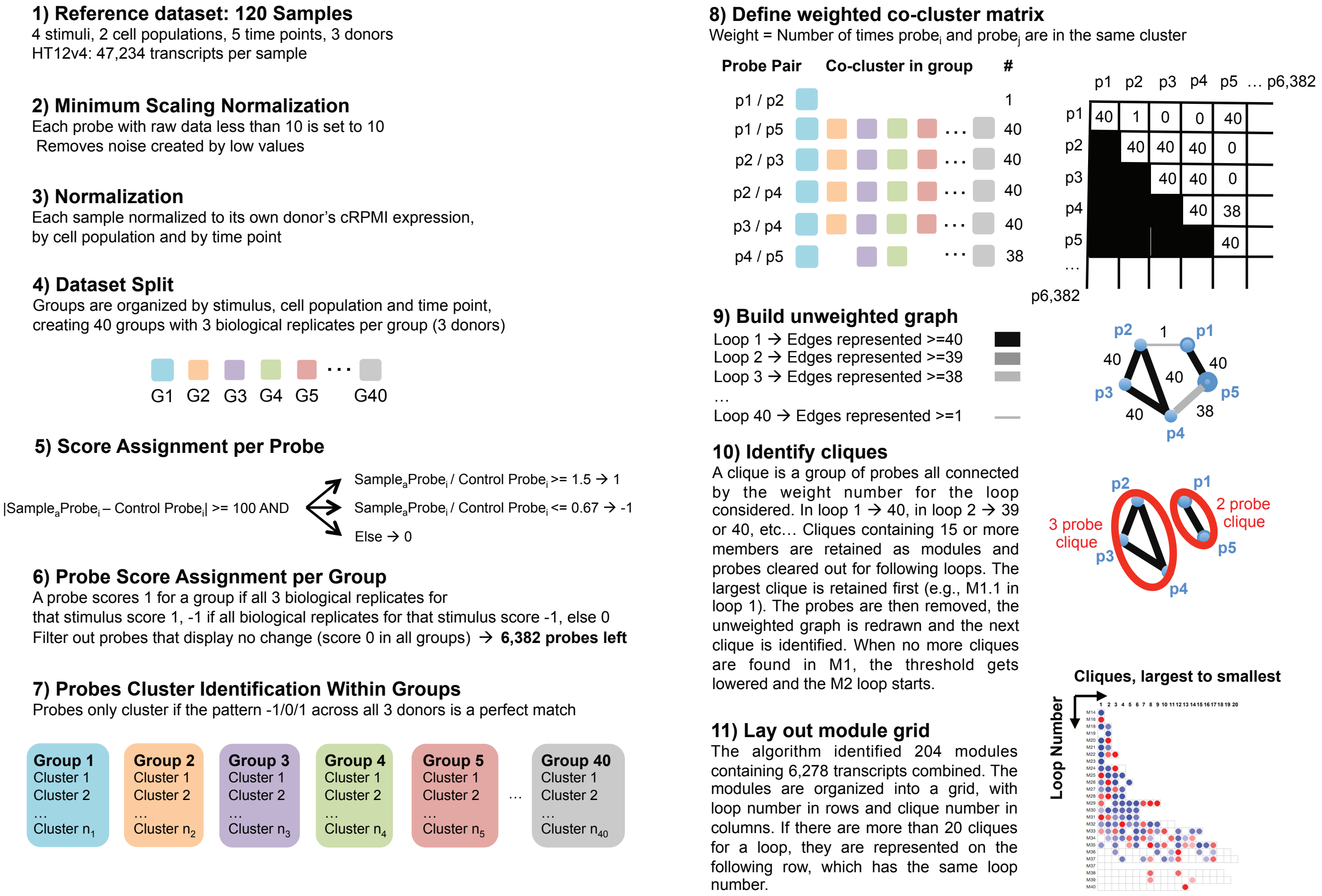

Supplementary Figure 2 - Module construction algorithm

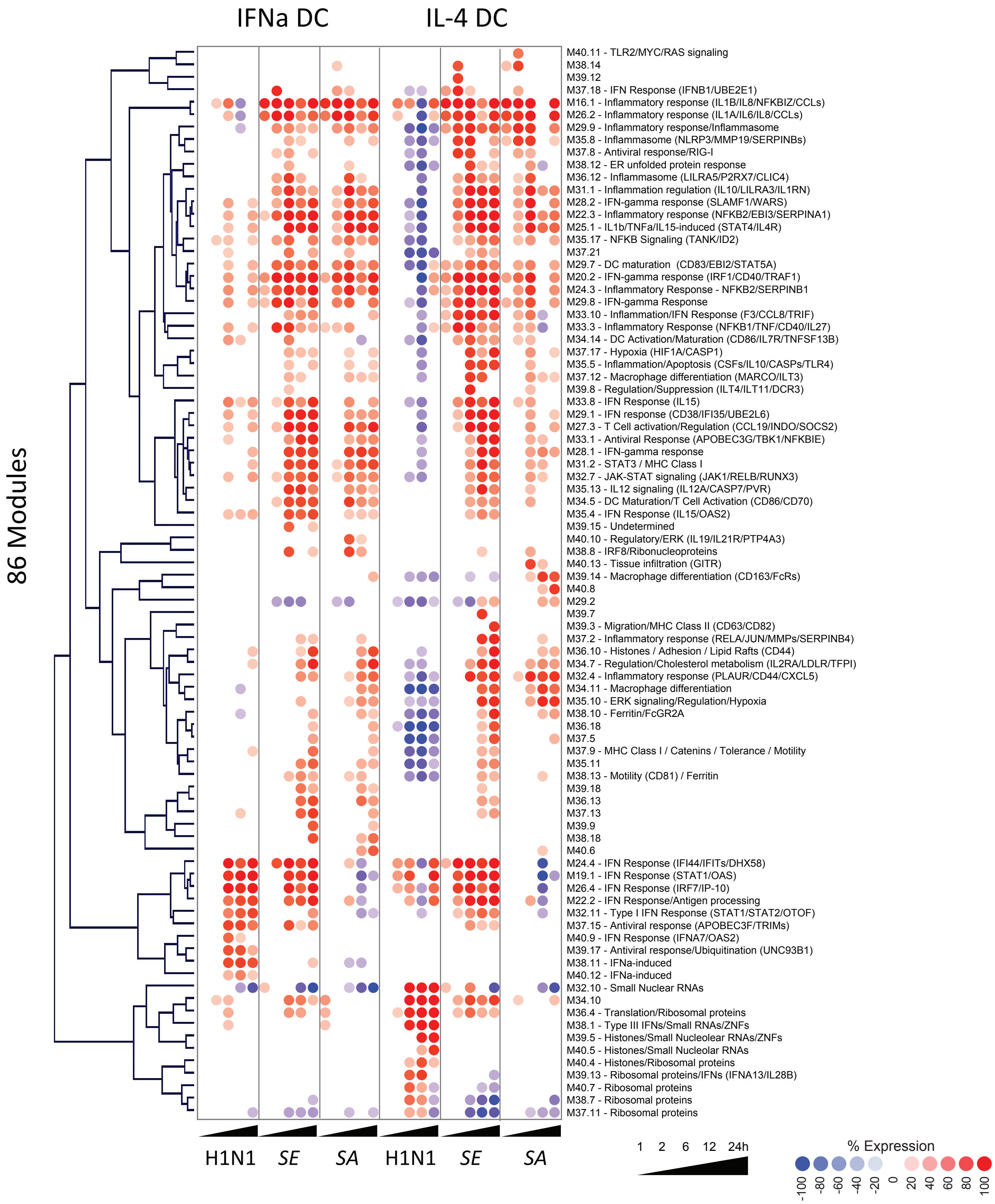

Supplementary Figure 3 - Heatmap representing the 86 modules overexpressed at least once (≥ 40%) in DC in response to pathogen challenge. Modules are hierarchically clustered (Pearson correlation). (H1N1: Influenza H1N1 Brisbane, SE: Heat-killed *S. enterica*, SA: Heat-killed *S. aureus*)

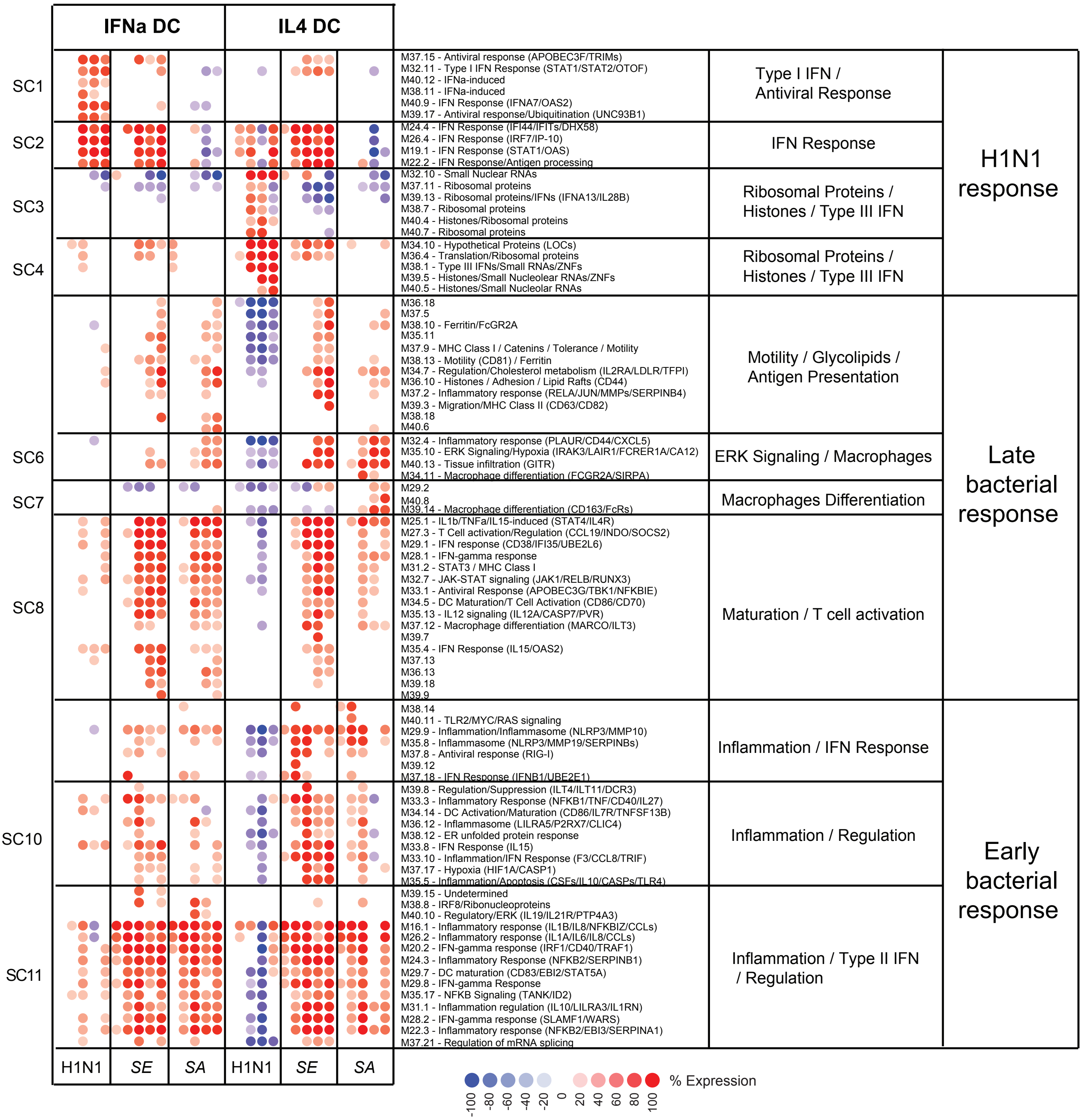

Supplementary Figure 4 - SOTA analysis identifies 11 module clusters defining the breadth of the modular transcriptional response of APC to H1N1, S. enterica and S. aureus in vitro over time. (H1N1: Influenza H1N1 Brisbane, SE: Heat-killed S. enterica, SA: Heat-killed S. aureus, SC: SOTA Cluster)

SC1: Interferon response

(IFNa DC - H1N1 - 6h)

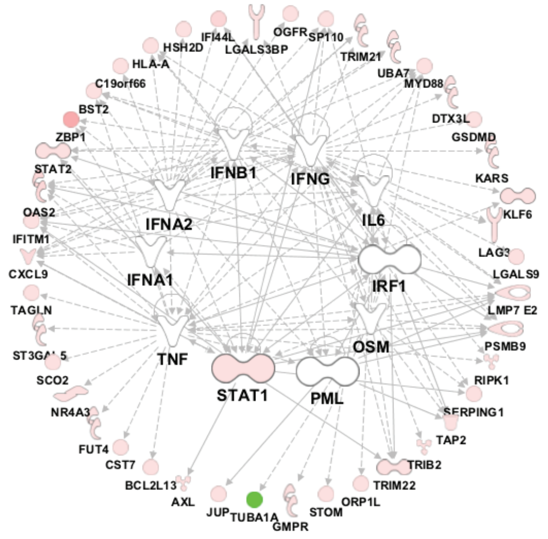

SC2: Interferon response

(IFNa DC - *S. enterica* - 6h)

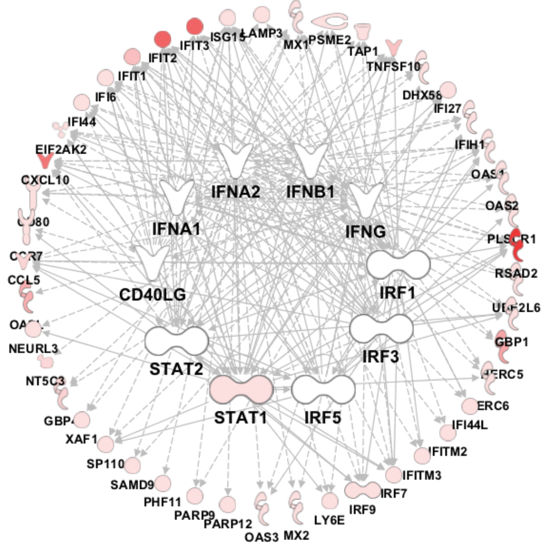

SC3: Ribosomal Proteins / Histones

(IL-4 DC - H1N1 - 6h)

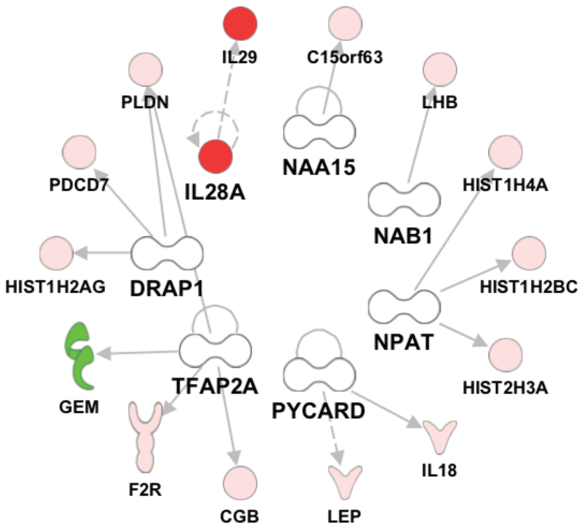

SC4: Histones / Type III IFN

(IL-4 DC – H1N1 – 6h)

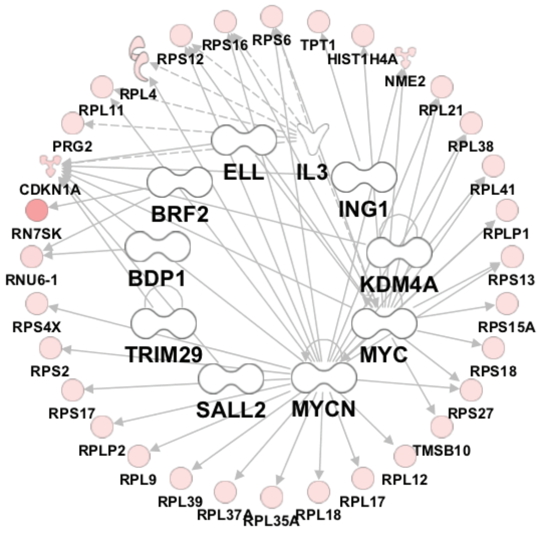

SC5: Motility / Glycolipids / Antigen Presentation

(IFNa DC - *S. enterica* - 24h)

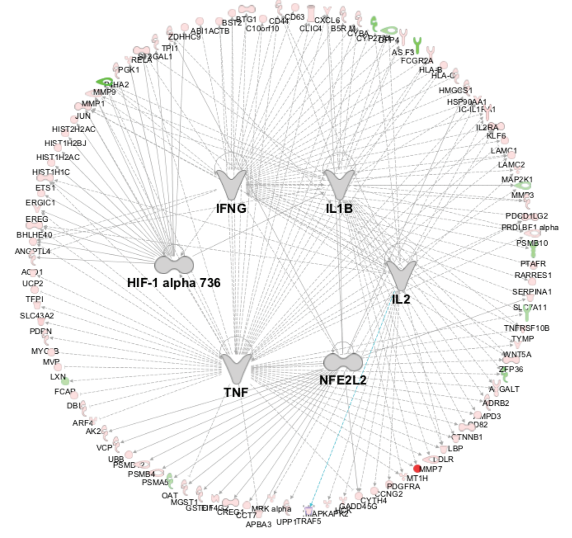

SC6: ERK Signaling / Macrophages

(IL-4 DC - *S. aureus* - 6h)

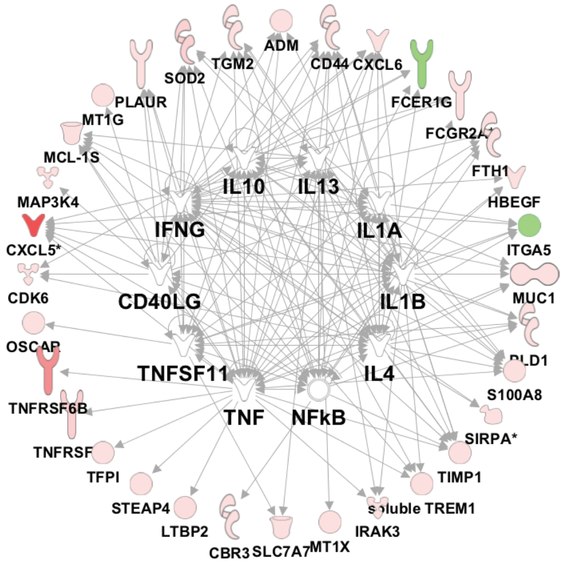

SC7: Macrophages Differentiation

(IL-4 DC – *S. aureus* - 24h)

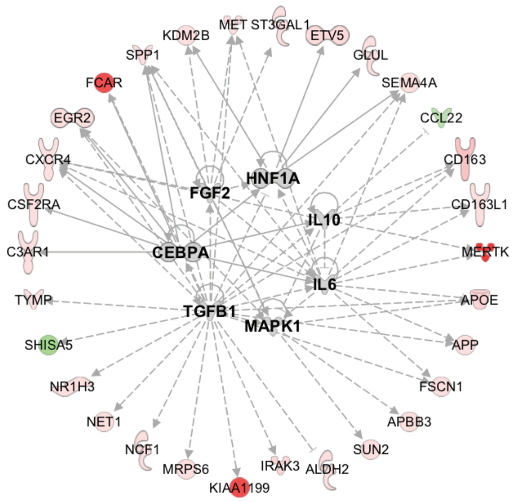

SC8: Maturation / T Cell Activation

(IFNa DC - *S. enterica* - 24h)

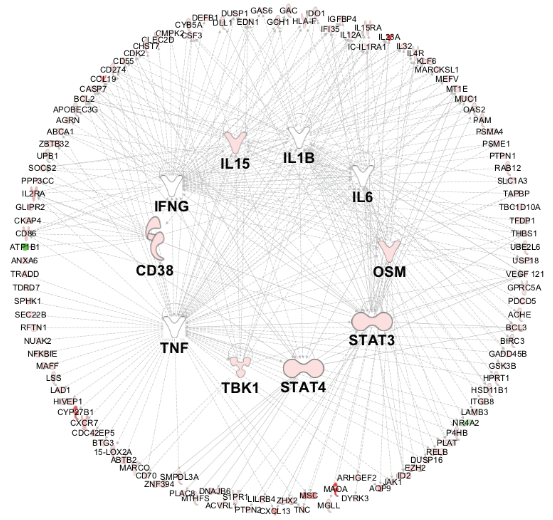

SC9: Inflammation / IFN Response

(IL-4 DC - *S. enterica* - 6h)

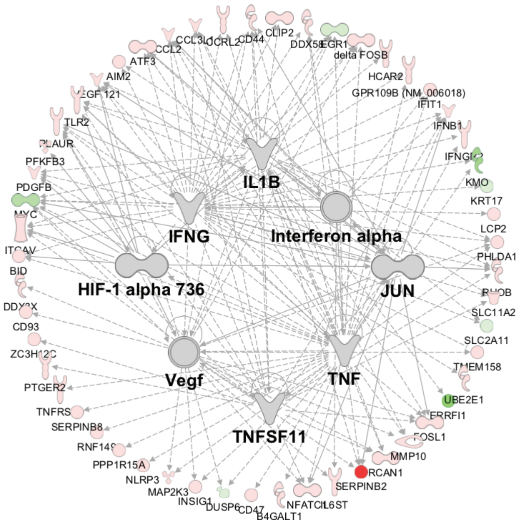

SC10: Inflammation / Regulation

(IL-4 DC - *S. enterica* - 6h)

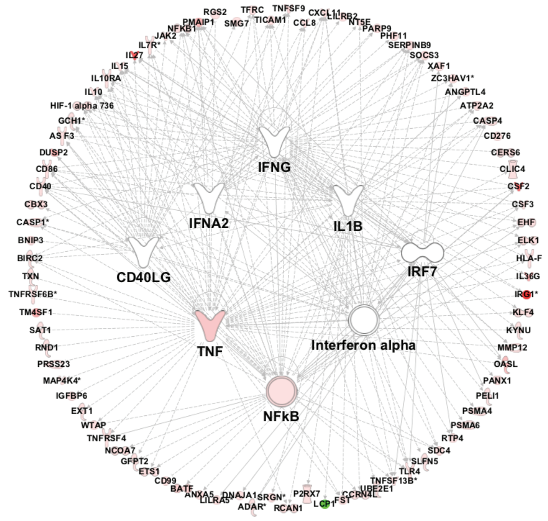

SC11: Inflammation / Regulation

(IFNa DC - *S. enterica* - 6h)

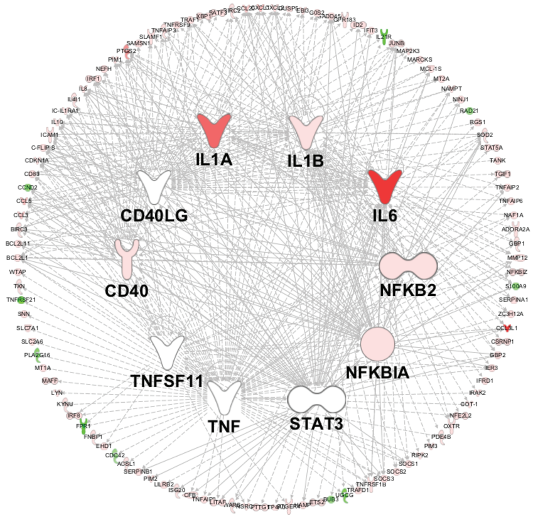

Supplementary Figure 5 - Ingenuity pathway analysis of the 11 clusters of modules identified by SOTA analysis. The molecules are colored according to their expression in the cell population, pathogen and time point specified in parentheses. The molecules in the center represent the in silico-predicted cytokines and transcriptional regulators up or downstream of the module transcripts.

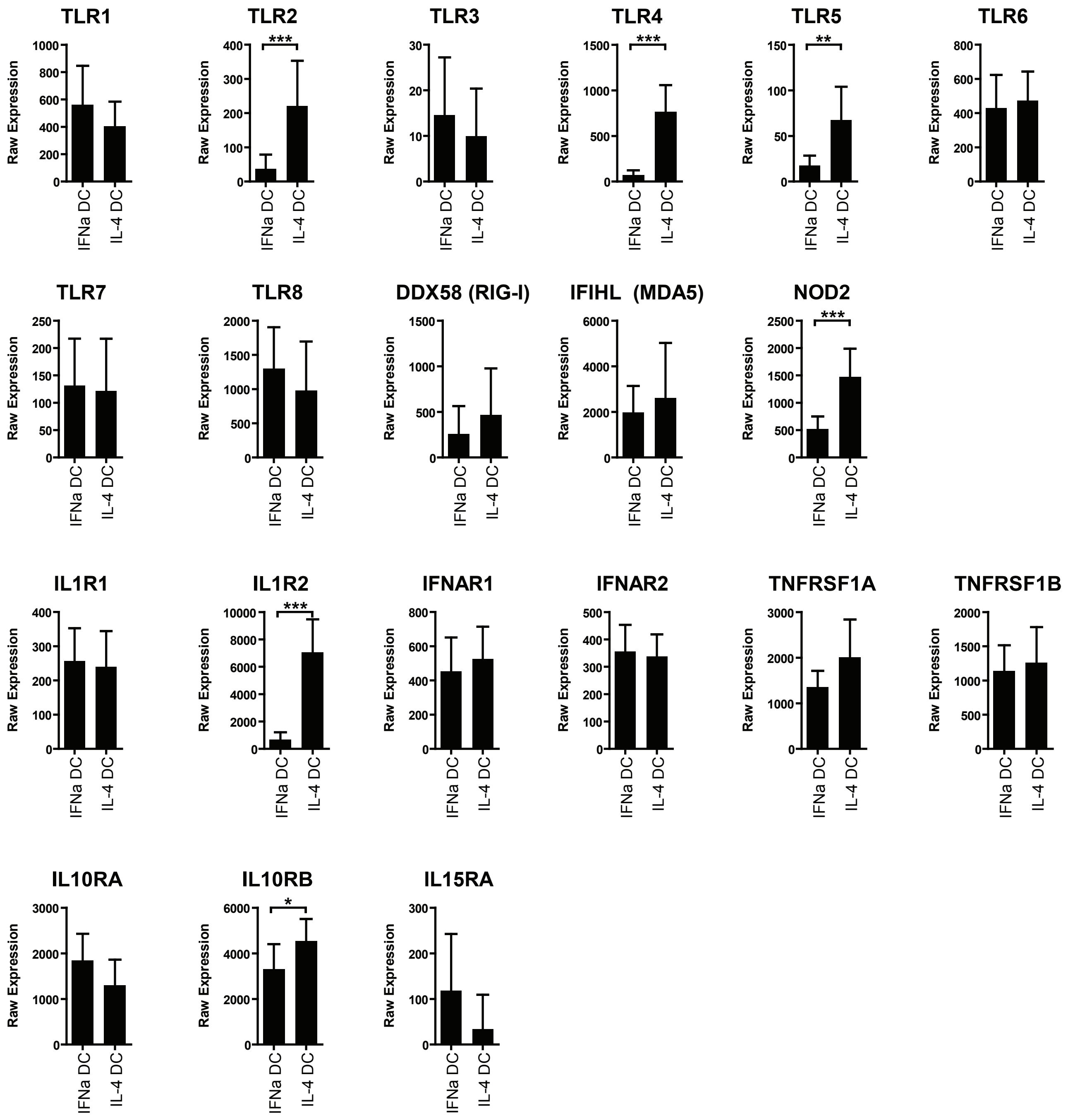

Supplementary Figure 6 - Analysis of transcriptional expression of PRRs and cytokine receptors in IFNα and IL-4 DC at baseline (6h in media alone). Raw data is displayed. Columns represent the mean of 9 biological replicates (9 independent donors). Error bars represent the standard deviation.

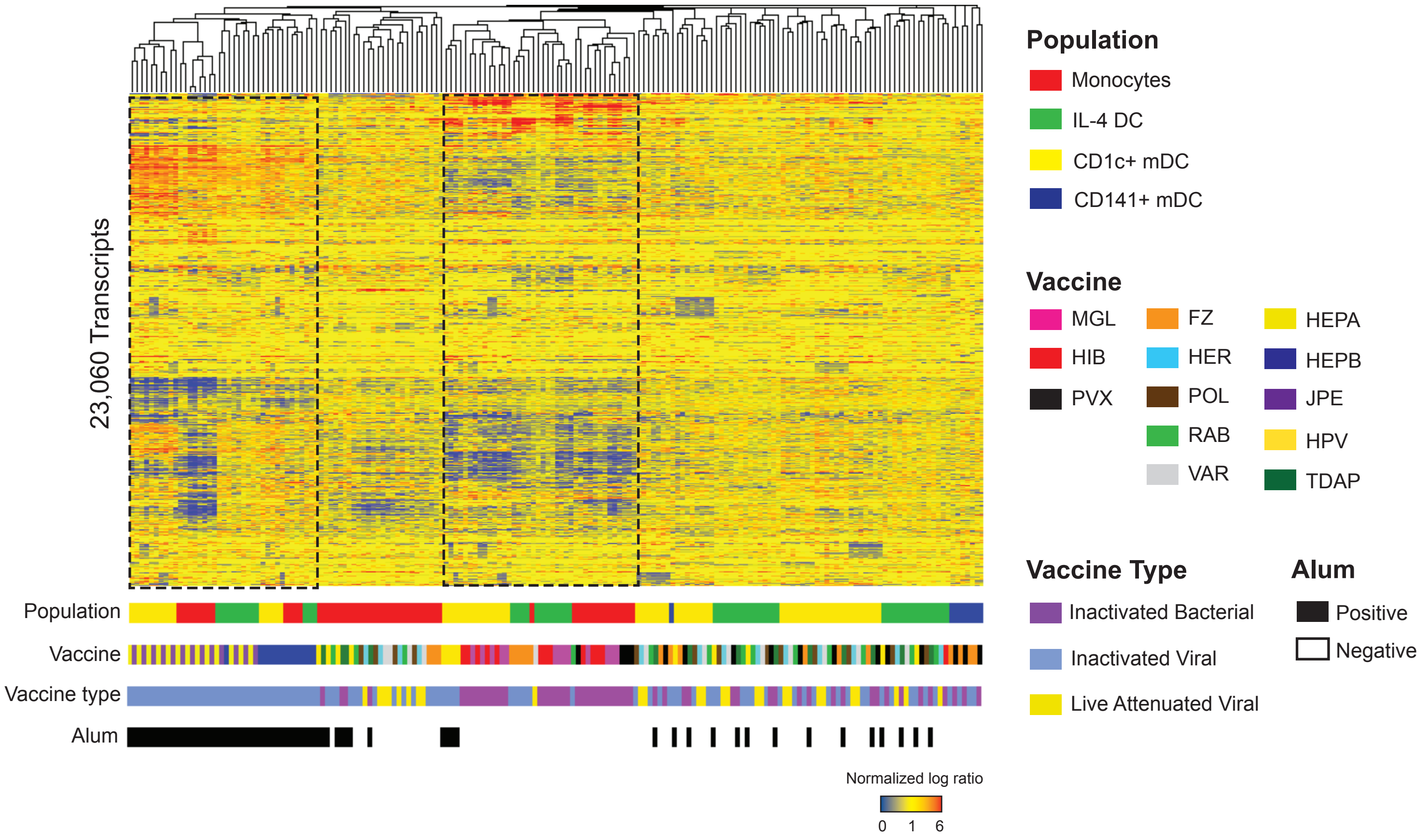

Supplementary Figure 7 - Unsupervised hierarchical clustering (Pearson correlation) of the 23,060 transcripts detected in monocytes, IL-4 DC, CD1c+ and CD141+ mDC stimulated with 13 vaccines for 6h. Samples are colored by cell population, vaccine, vaccine type and alum content.

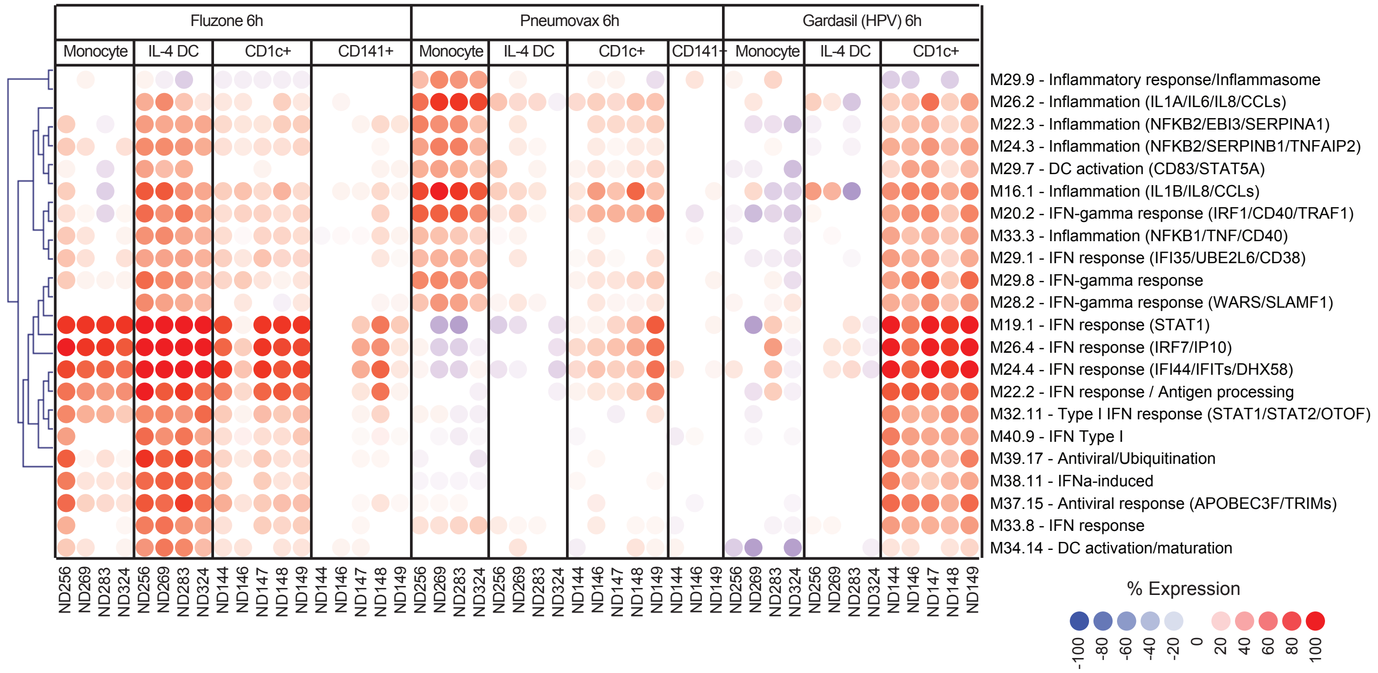

Supplementary Figure 8 - Individual modular fingerprints of monocytes, IL-4 DC, CD1c+ and CD141+ mDC in response to 6h activation with Fluzone (influenza), Pneumovax (pneumococcus) or Gardasil (HPV).

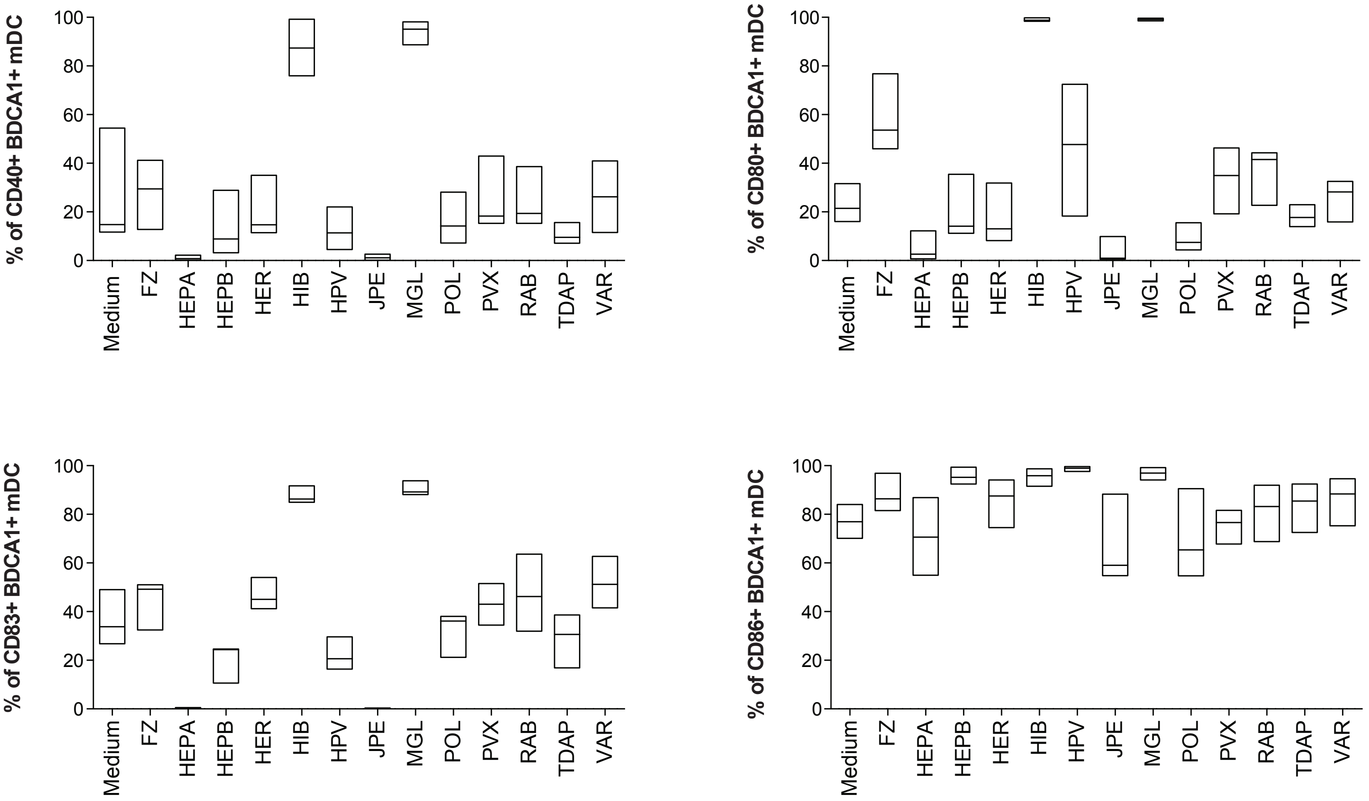

Supplementary Figure 9 - Expression of CD40, CD80, CD83, and CD86 on CD1c+ mDC after 24h stimulation with vaccines in vitro. Floating bars (min to max, line at median) representing the percent of live CD1c+ mDC positive for CD40, CD80, CD83 and CD86.

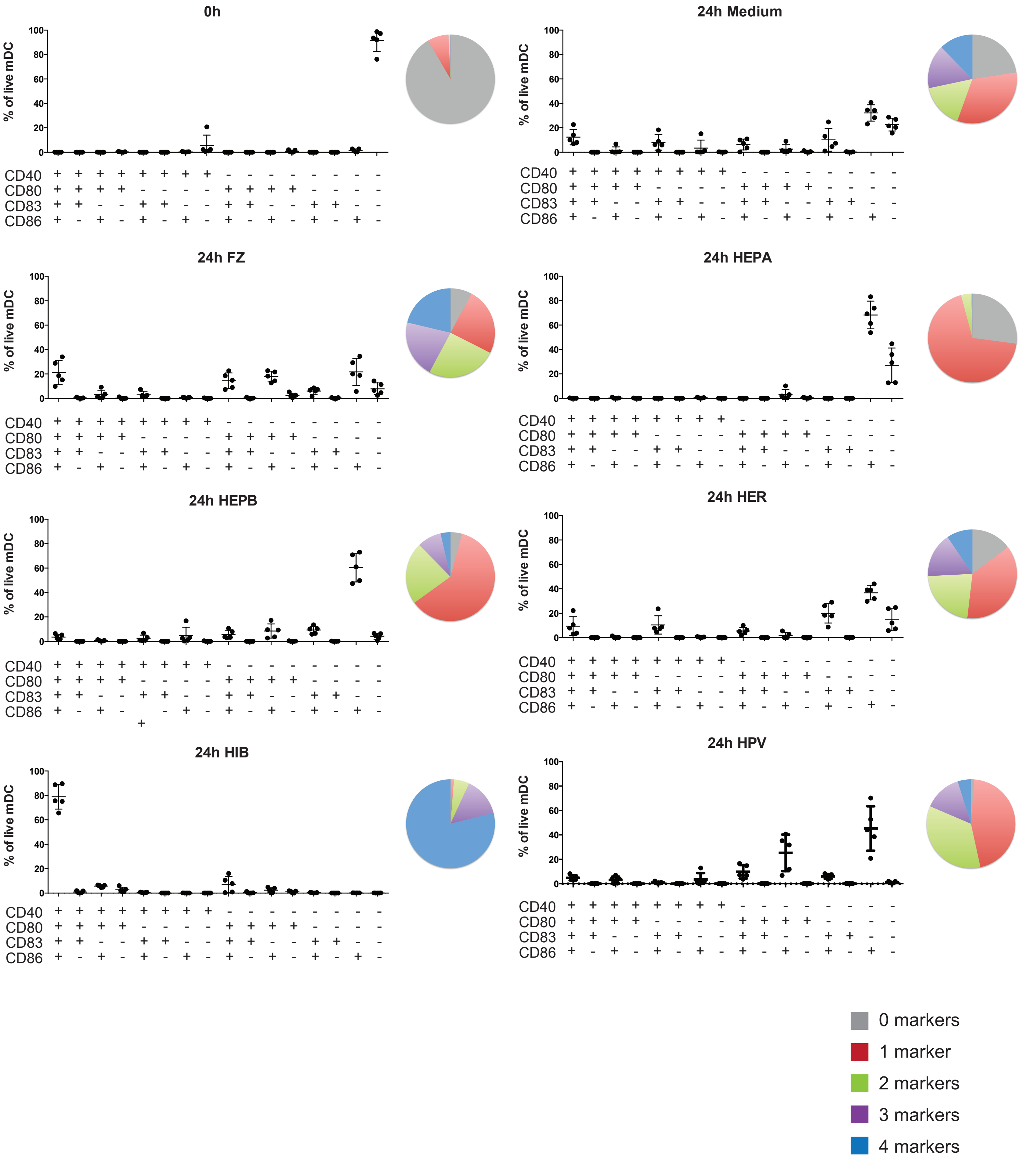
0h
24h Medium
24h FZ
24h HEPA
24h HEPB
24h HER
24h HIB
24h HPV
% of live mDC
CD40
CD80
CD83
CD86
0 markers
1 marker
2 markers
3 markers
4 markers

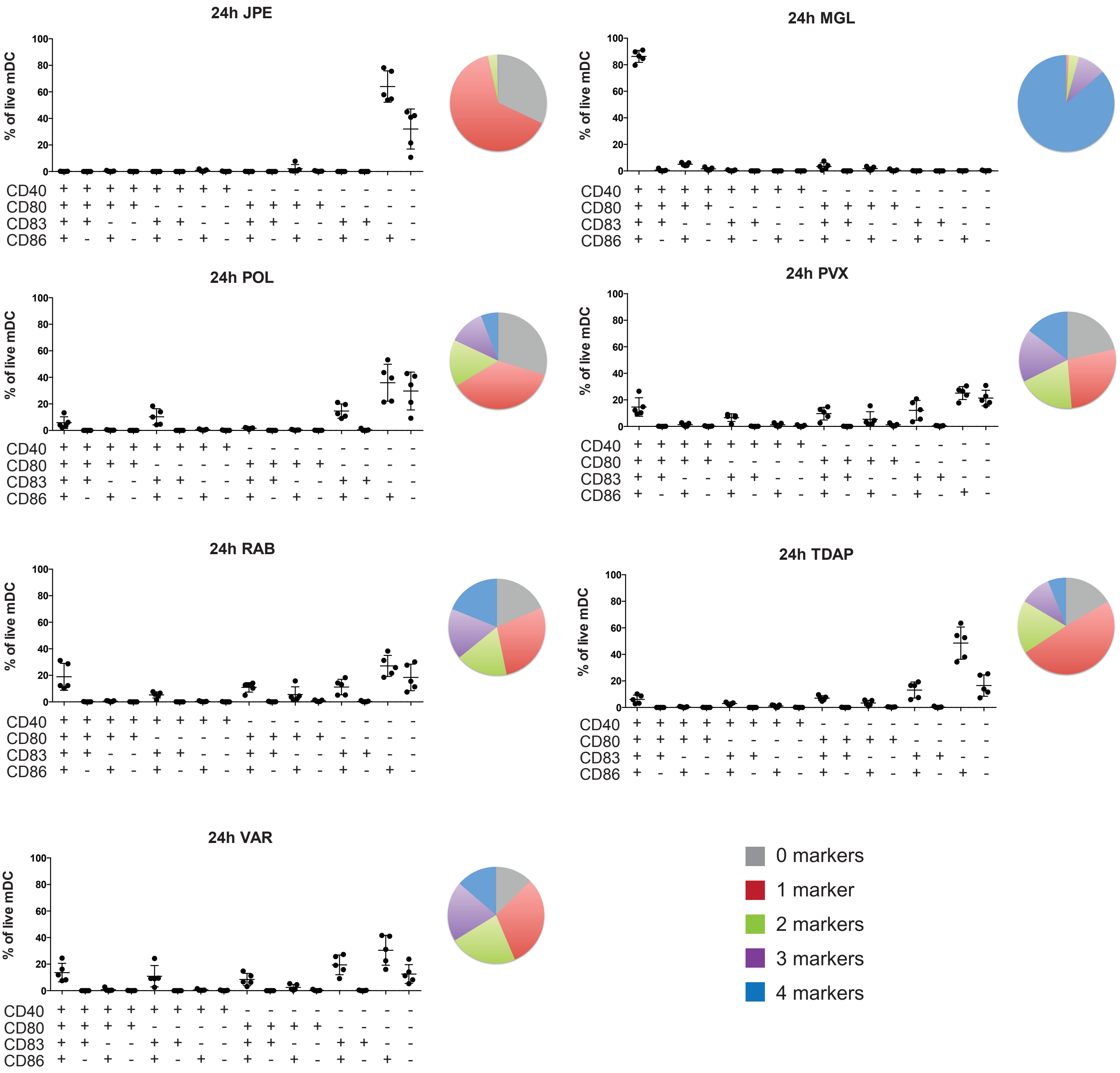

Supplementary Figure 10 - Activation markers induced by vaccines in CD1c+ mDC after 24h stimulation. Boolean gating representing the expression of CD40, CD80, CD83 and CD86 for 5 healthy donors. Pie charts represent the proportion of live mDC expressing 0, 1, 2, 3 or all 4 activation markers.

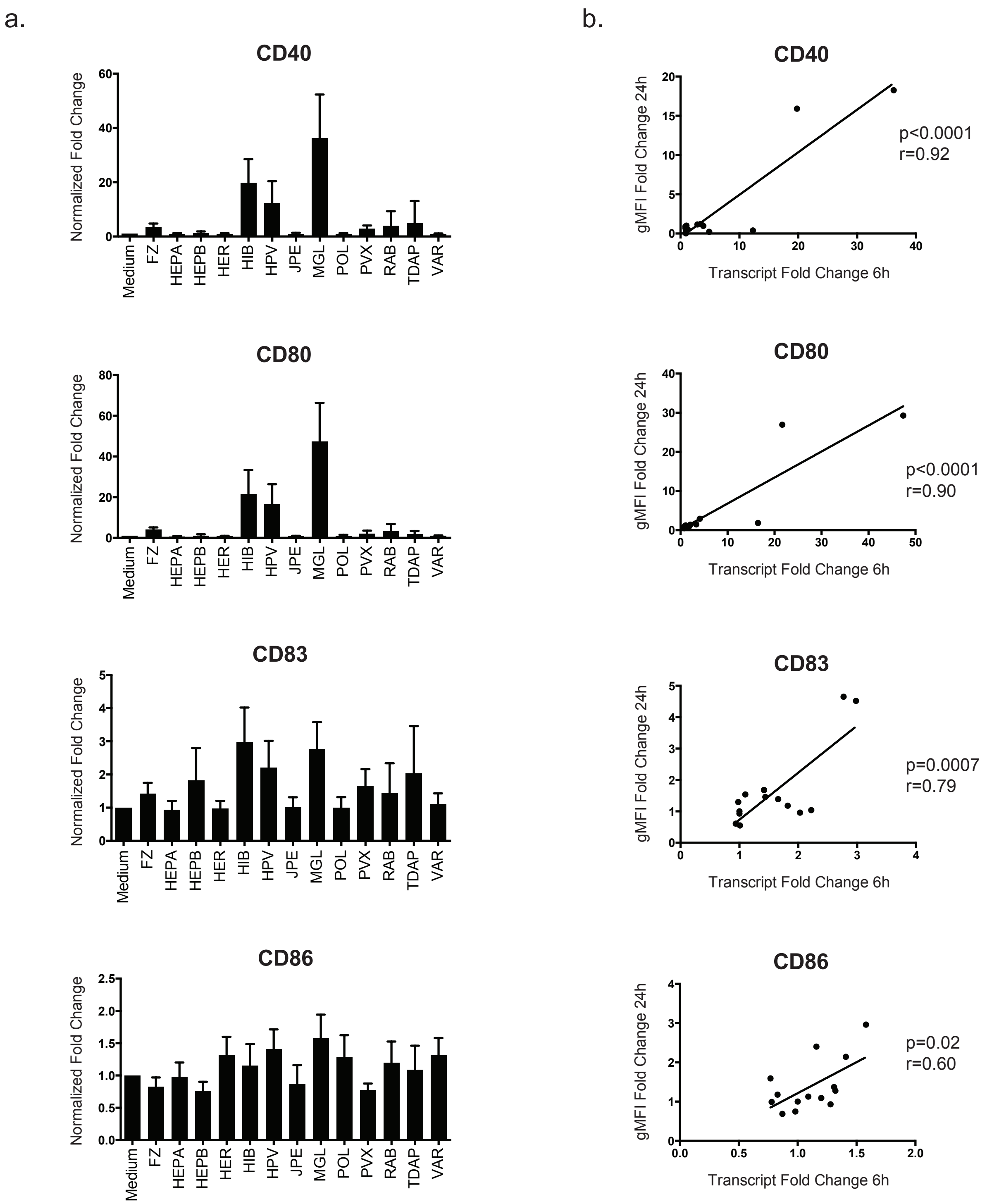

Supplementary Figure 11 - Changes in CD1c+ mDC maturation markers at 24h in response to vaccines correlate with changes in transcript expression at 6h. a. Bar charts representing the normalized fold change of CD40, CD80, CD83 and CD86 transcripts as compared to 6h medium for 5 donors. Error bars represent the standard deviation. **b.** X-Y charts representing the transcript fold change on the X-axis (normalized to 6h medium) and the change in gMFI measured by FACS at 24h (normalized to 24h medium) for the four markers. Linear regression lines were drawn and correlations were calculated (Pearson).

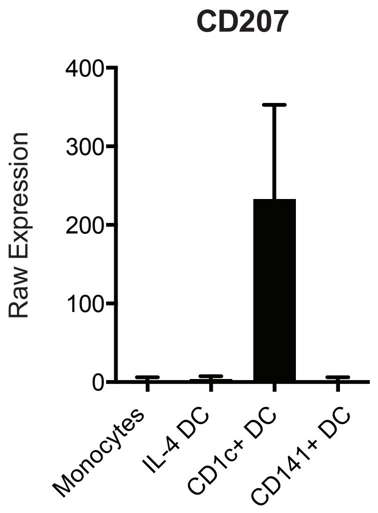

Supplementary Figure 12 - Raw transcriptional expression of langerin (CD207) in APC subsets

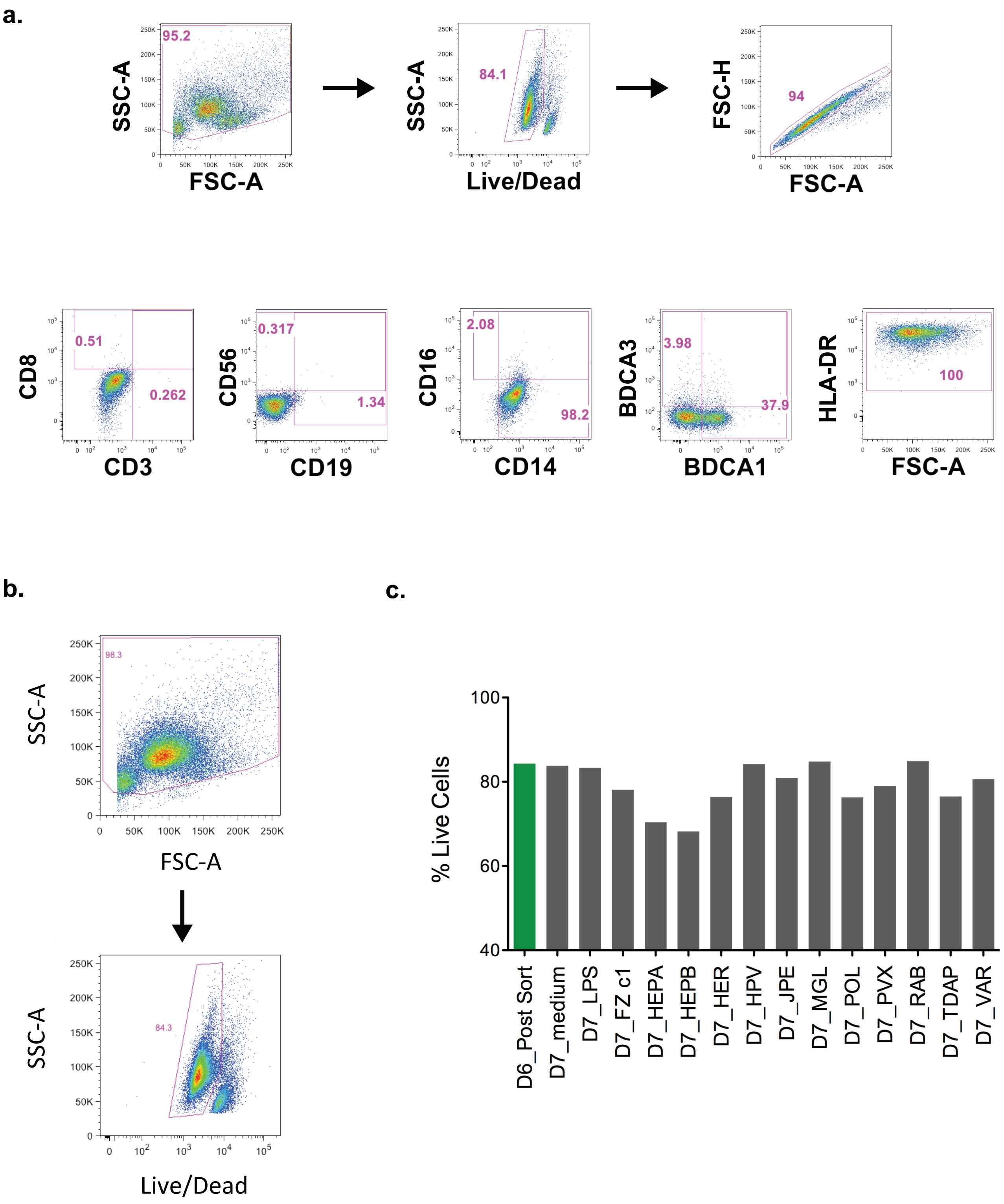

Supplementary Figure 13 - IL-4 DC purity panel and viability analyses. **a.** Purity panel. b. Gating on viability dye. **c.** Viability of IL-4 DCstimulated for 24h with vaccines

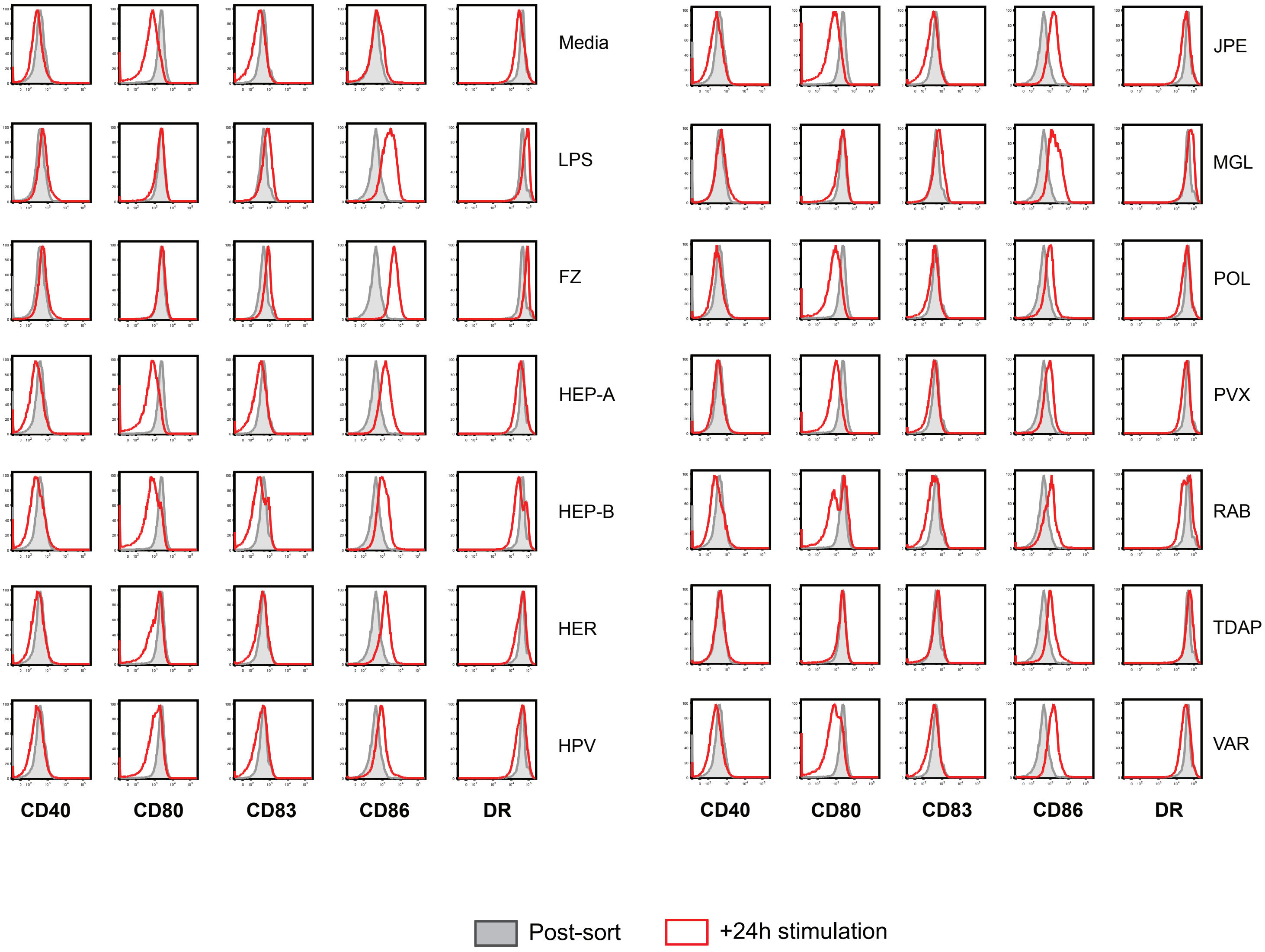

Supplementary Figure 14 - Induction of surface maturation markers CD40, CD80, CD83, CD86 and HLA-DR in IL-4 DC stimulated with vaccines for 24h

Supplementary Table 1 - Module Annotations

#	Module ID	Annotation	Round	Column	Transcripts Count
1	M14.1		14	1	15
2	M16.1	Inflammatory response (IL1B/IL8/NFKBIZ/CCLs)	16	1	16
3	M18.1	IL10-induced	18	1	18
4	M18.2	CD1 Family/C-type Lectins (CD1s/ASGPR /CLEC10A)	18	2	16
5	M19.1	IFN Response (STAT1/OAS)	19	1	15
6	M19.2	IL10-induced (CTSB)	19	2	15
7	M20.1		20	1	19
8	M20.2	IFN-gamma response (IRF1/CD40/TRAF1)	20	2	18
9	M21.1		21	1	22
10	M21.2	Chemokines/FcRs	21	2	16
11	M22.1		22	1	20
12	M22.2	IFN Response/Antigen processing	22	2	16
13	M22.3	Inflammatory response (NFKB2/EBI3/SERPINA1)	22	3	16
14	M23.1		23	1	23
15	M24.1		24	1	26
16	M24.2		24	2	20
17	M24.3	Inflammatory Response (NFKB2/SERPINB1)	24	3	19
18	M24.4	IFN Response (IFI44/IFITs/DHX58)	24	4	17
19	M25.1	IL1b/TNFa/IL15-induced (STAT4/IL4R)	25	1	23
20	M25.2		25	2	17
21	M25.3		25	3	15
22	M25.4		25	4	15
23	M26.1		26	1	25
24	M26.2	Inflammatory response (IL1A/IL6/IL8/CCLs)	26	2	17
25	M26.3		26	3	16
26	M26.4	IFN Response (IRF7/IP-10)	26	4	16
27	M26.5		26	5	15
28	M27.1		27	1	18
29	M27.2		27	2	16
30	M27.3	T Cell activation/Regulation (CCL19/INDO/SOCS2)	27	3	16
31	M27.4		27	4	15
32	M28.1	IFN-gamma response	28	1	19
33	M28.2	IFN-gamma response (SLAMF1/WARS)	28	2	17
34	M28.3		28	3	17
35	M28.4		28	4	17
36	M29.1	IFN response (CD38/IFI35/UBE2L6)	29	1	26
37	M29.2		29	2	20
38	M29.3		29	3	18
39	M29.4		29	4	18
40	M29.5		29	5	16
41	M29.6		29	6	15
42	M29.7	DC maturation (CD83/EBI2/STAT5A)	29	7	15
43	M29.8	IFN-gamma Response	29	8	15
44	M29.9	Inflammation/Inflammasome (NLRP3/MMP10)	29	9	15
45	M30.1		30	1	26
46	M30.2		30	2	23
47	M30.3		30	3	21
48	M30.4		30	4	18

49	M30.5		30	5	17
50	M30.6		30	6	15
51	M31.1	Inflammation regulation (IL10/LILRA3/IL1RN)	31	1	26
52	M31.2	STAT3 / MHC Class I	31	2	22
53	M31.3	IL10-induced - CTSA/RNASE4	31	3	21
54	M31.4		31	4	19
55	M31.5		31	5	18
56	M31.6		31	6	15
57	M32.1		32	1	38
58	M32.10	Small Nuclear RNAs	32	10	17
59	M32.11	Type I IFN Response (STAT1/STAT2/OTOF)	32	11	15
60	M32.2		32	2	33
61	M32.3		32	3	25
62	M32.4	Inflammatory response (PLAUR/CD44/CXCL5)	32	4	24
63	M32.5		32	5	21
64	M32.6		32	6	21
65	M32.7	JAK-STAT signaling (JAK1/RELB/RUNX3)	32	7	20
66	M32.8		32	8	19
67	M32.9		32	9	19
68	M33.1	Antiviral Response (APOBEC3G/TBK1/NFKBIE)	33	1	35
69	M33.10	Inflammation/IFN Response (F3/CCL8/TRIF)	33	10	19
70	M33.11		33	11	19
71	M33.12		33	12	17
72	M33.13		33	13	15
73	M33.14	Hypothetical/Ribosomal Proteins	33	14	15
74	M33.15		33	15	15
75	M33.2		33	2	31
76	M33.3	Inflammatory Response (NFKB1/TNF/CD40/IL27)	33	3	29
77	M33.4		33	4	27
78	M33.5		33	5	25
79	M33.6		33	6	25
80	M33.7		33	7	22
81	M33.8	IFN Response (IL15)	33	8	20
82	M33.9		33	9	19
83	M34.1		34	1	44
84	M34.10	Hypothetical Proteins (LOCs)	34	10	20
85	M34.11	Macrophage differentiation (FCGR2A/SIRPA)	34	11	18
86	M34.12		34	12	17
87	M34.13	IL10-induced	34	13	16
88	M34.14	DC Activation/Maturation (CD86/IL7R/TNFSF13B)	34	14	15
89	M34.2		34	2	38
90	M34.3		34	3	36
91	M34.4		34	4	36
92	M34.5	DC Maturation/T Cell Activation (CD86/CD70)	34	5	25
93	M34.6		34	6	24
94	M34.7	Regulation/Cholesterol metabolism (IL2RA/LDLR/TFPI)	34	7	21
95	M34.8		34	8	20
96	M34.9		34	9	20
97	M35.1		35	1	52
98	M35.10	ERK Signaling/Regulation/Hypoxia (IRAK3/LAIR1/FCRER1A/CA	35	10	21
99	M35.11		35	11	20
100	M35.12		35	12	19
101	M35.13	IL12 signaling (IL12A/CASP7/PVR)	35	13	18

102	M35.14		35	14	18
103	M35.15		35	15	17
104	M35.16		35	16	16
105	M35.17	NFKB Signaling (TANK/ID2)	35	17	15
106	M35.2		35	2	42
107	M35.3	Hypothetical/Ribosomal Proteins	35	3	39
108	M35.4	IFN Response (IL15/OAS2)	35	4	30
109	M35.5	Inflammation/Apoptosis (CSFs/IL10/CASPs/TLR4)	35	5	30
110	M35.6		35	6	28
111	M35.7		35	7	24
112	M35.8	Inflammasome (NLRP3/MMP19/SERPINBs)	35	8	23
113	M35.9	Hypothetical Proteins (LOCs)	35	9	23
114	M36.1		36	1	86
115	M36.10	Histones / Adhesion / Lipid Rafts (CD44)	36	10	23
116	M36.11		36	11	21
117	M36.12	Inflammasome (LILRA5/P2RX7/CLIC4)	36	12	21
118	M36.13		36	13	18
119	M36.14	Mitochondrial Proteins	36	14	18
120	M36.15		36	15	17
121	M36.16		36	16	17
122	M36.17		36	17	17
123	M36.18		36	18	15
124	M36.19		36	19	15
125	M36.2		36	2	38
126	M36.3		36	3	35
127	M36.4	Translation/Ribosomal proteins	36	4	35
128	M36.5		36	5	31
129	M36.6		36	6	31
130	M36.7		36	7	29
131	M36.8	Hypothetical/Ribosomal proteins (LOCs)	36	8	27
132	M36.9		36	9	25
133	M37.1		37	1	132
134	M37.10		37	10	27
135	M37.11	Ribosomal proteins	37	11	27
136	M37.12	Macrophage differentiation (MARCO/ILT3)	37	12	25
137	M37.13		37	13	24
138	M37.14		37	14	22
139	M37.15	Antiviral response (APOBEC3F/TRIMs)	37	15	22
140	M37.16	ATP Synthases /Ribosomal Proteins	37	16	22
141	M37.17	Hypoxia (HIF1A/CASP1)	37	17	20
142	M37.18	IFN Response (IFNB1/UBE2E1)	37	18	19
143	M37.19		37	19	17
144	M37.2	Inflammatory response (RELA/JUN/MMPs/SERPINB4)	37	2	69
145	M37.20		37	20	17
146	M37.21	Regulation of mRNA splicing	37	21	16
147	M37.22		37	22	16
148	M37.3		37	3	56
149	M37.4	Hypothetical/Ribosomal proteins	37	4	42
150	M37.5		37	5	38
151	M37.6		37	6	37
152	M37.7		37	7	33
153	M37.8	Antiviral response (RIG-I)	37	8	33
154	M37.9	MHC Class I / Catenins / Tolerance / Motility	37	9	28

155	M38.1	Type III IFNs/Small RNAs/ZNFs	38	1	159
156	M38.10	Ferritin/FcGR2A	38	10	24
157	M38.11	IFNa-induced	38	11	23
158	M38.12	ER unfolded protein response	38	12	21
159	M38.13	Motility (CD81) / Ferritin	38	13	20
160	M38.14		38	14	19
161	M38.15		38	15	18
162	M38.16		38	16	17
163	M38.17		38	17	17
164	M38.18		38	18	15
165	M38.2		38	2	136
166	M38.3		38	3	59
167	M38.4		38	4	54
168	M38.5		38	5	43
169	M38.6		38	6	36
170	M38.7	Ribosomal proteins	38	7	29
171	M38.8	IRF8/Ribonucleoproteins	38	8	27
172	M38.9		38	9	24
173	M39.1		39	1	307
174	M39.10		39	10	44
175	M39.11		39	11	37
176	M39.12		39	12	35
177	M39.13	Ribosomal proteins/IFNs (IFNA13/IL28B)	39	13	34
178	M39.14	Macrophage differentiation (CD163/FcRs)	39	14	32
179	M39.15	Undetermined	39	15	28
180	M39.16		39	16	22
181	M39.17	Antiviral response/Ubiquitination (UNC93B1)	39	17	19
182	M39.18		39	18	19
183	M39.19		39	19	18
184	M39.2		39	2	121
185	M39.3	Migration/MHC Class II (CD63/CD82)	39	3	94
186	M39.4		39	4	83
187	M39.5	Histones/Small Nucleolear RNAs/ZNFs	39	5	72
188	M39.6	Ribosomal proteins	39	6	66
189	M39.7		39	7	64
190	M39.8	Regulation/Suppression (ILT4/ILT11/DCR3)	39	8	53
191	M39.9		39	9	46
192	M40.1		40	1	177
193	M40.10	Regulatory/ERK (IL19/IL21R/PTP4A3)	40	10	20
194	M40.11	TLR2/MYC/RAS signaling	40	11	18
195	M40.12	IFNa-induced	40	12	15
196	M40.13	Tissue infiltration (GITR)	40	13	15
197	M40.2		40	2	141
198	M40.3		40	3	120
199	M40.4	Histones/Ribosomal proteins	40	4	40
200	M40.5	Histones/Small Nucleolar RNAs	40	5	36
201	M40.6		40	6	30
202	M40.7	Ribosomal proteins	40	7	29
203	M40.8		40	8	26
204	M40.9	IFN Response (IFNA7/OAS2)	40	9	25

Supplementary Table 2 - 11 clusters of modules identified by SOTA

Cluster #	Cluster Annotation	Module ID	Module Annotation
SC1	Interferon Response Antiviral Response / IFNa DC only	M37.15	Antiviral Response - APOBEC3F/TRIMs/TAP2
		M32.11	IFN Response - STAT1/STAT2/OTOF
		M40.12	IFNa-induced
		M40.9	IFN Response - IFNA7/OAS2/SP140
		M38.11	IFNa-induced
		M39.17	Antiviral/Ubiquitination - UNC93B1/SP110
SC2	Interferon Response Antiviral & Antibacterial / IFNa and IL-4 DC	M24.4	IFN Response - IFI44/IFITs/DHX58
		M26.4	IFN Response - IRF7/IP-10
		M19.1	IFN Response - STAT1/OAS
		M22.2	IFN Response / Antigen Processing
SC3	Ribosomal Proteins / Histones Antiviral / IL-4 DC only	M32.10	Small Nuclear RNAs
		M37.11	Ribosomal Proteins
		M39.13	Ribosomal Proteins / IFNs - IFNA13 / IL28B
		M38.7	Ribosomal Proteins
		M40.4	Histones / Ribosomal Proteins
		M40.7	Ribosomal Proteins
SC4	Histones / Type III Interferons Antiviral / IL-4 DC only	M34.10	
		M36.4	Transcription/Translation - Ribosomal Proteins
		M38.1	Type III IFNs/Small RNAs/ZNFs
		M39.5	Histones/Small Nucleolear RNAs/ZNFs
		M40.5	Histones/Small Nucleolar RNAs
SC5	Motility / Glycolipids / Antigen Presentation<br><br>Late Antibacterial / IFNa and IL-4 DC	M36.18	
		M37.5	
		M38.10	Ferritin/FcGR2A
		M35.11	
		M37.9	MHC Class I / Catenins / Tolerance / Motility
		M38.13	Motility (CD81) / Ferritin
		M34.7	Regulation/Cholesterol metabolism - IL2RA/LDLR/TFPI
		M36.10	Histones / Adhesion / Lipid Rafts (CD44)
		M37.2	Inflammatory Response - RELA / JUN / MMPs / SERPINB4
		M39.3	Migration / MHC Class II (CD63 / CD82)
		M38.18	
		M40.6	
SC6	ERK Signaling / Macrophages Late Antibacterial / IFNa and IL-4 DC	M32.4	Inflammatory Response - PLAUR/CD44/CXCL5
		M35.10	ERK Signaling/Regulation/Hypoxia - IRAK3/LAIR1/FCRER1A
		M40.13	Tissue infiltration - GITR
		M34.11	Macrophage differentiation - FCGR2A/SIRPA
SC7	Macrophage Differentiation Late Antibacterial / IL-4 DC only	M29.2	
		M40.8	
		M39.14	Macrophage differentiation - CD163/FcRs
SC8	Maturation / T Cell Activation Late Antibacterial / IFNa and IL-4 DC	M25.1	IL1b TNFa IL15-induced - STAT4/IL4R/LILRA3
		M27.3	T Cell Activation/Tolerance - CCL19/INDO/CD274/SOCS2
		M29.1	IFN Response - CD38/IFI35/UBE2L6
		M28.1	
		M31.2	STAT3 / MHC Class I
		M32.7	JAK1/RELB/RUNX3
		M33.1	Antiviral Response - APOBEC3G/TBK1/NFKBIE
		M34.5	DC Maturation/T Cell Activation - CD86/CD70
		M35.13	IL12A/CASP7/PVR

	DC	M37.12	Macrophage differentiation - MARCO/ILT3
		M39.7	
		M35.4	IFN Response - IL15/OAS2
		M37.13	
		M36.13	
		M39.18	
		M39.9	
SC9	Inflammation / Interferon Response<br><br>Early Antibacterial / IL-4 DC only	M38.14	
		M40.11	TLR2/MYC/RAS signaling
		M29.9	Inflammatory Response - NLRP3/MMP10/SERPINB2
		M35.8	Inflammatory Response - NLRP3/MMP19/SERPINBs
		M37.8	Antiviral - RIG-I
		M39.12	
		M37.18	IFN Response - IFNB1/UBE2E1
SC10	Inflammation / Regulation<br><br>Early Antibacterial / IFNa and IL-4 DC	M39.8	Regulation/Suppression - ILT4/ILT11/DCR3
		M33.3	Inflammatory Response - NFKB1/TNF/CD40/IL27
		M34.14	DC Activation/Maturation - CD86/IL7R/HLA-F/TNFSF13B
		M36.12	Inflammasome-related - LILRA5/P2RX7/SOCS3/CLIC4
		M38.12	
		M33.8	IFN Response - IL15
		M33.10	Inflammatory/IFN Response - F3/CCL8/TRIF
		M37.17	Hypoxia/Apoptosis - HIF1A/CASP1
		M35.5	Inflammation/Apoptosis - CSFs/IL10/CASPs/TLR4
SC11	Inflammation / Regulation<br><br>Early Antibacterial / IFNa and IL-4 DC	M39.15	
		M38.8	IRF8/Ribonucleoproteins
		M40.10	Regulatory/ERK - IL19/IL21R/PTP4A3
		M16.1	Inflammatory Response - IL1B/IL8/NFKBIZ/CCLs
		M26.2	Inflammatory Response - IL1A/IL6/IL8/CCLs
		M20.2	IFN-gamma Response - IRF1/CD40/TRAF1
		M24.3	Inflammatory Response - NFKB2/SERPINAB1/TNFAIP2
		M29.7	CD83/EBI2/STAT5A
		M29.8	IFN-gamma Response
		M35.17	NFKB Signaling - TANK/ID2
		M31.1	Inflammation Regulation - IL10/LILRA3/IL1RN
		M28.2	SLAMF1/TNFRSF1B/WARS
		M22.3	Inflammatory Response - NFKB2/EBI3/SERPINA1
		M37.21	

Supplementary Table 3 - Stimuli Summary

Category	Ligand	Category	Source	Dose	Target
TLR Ligands & Cytoplasmic Receptors	PAM3-CSK4	Bacterial cell wall component	Invivogen	200ng/mL	TLR1/2
	Poly I:C	Synthetic analog of dsRNA	Invivogen	10ug/mL	TLR3
	LPS	Gram-negative bacterial cell wall component	Invivogen	100ng/mL	TLR4
	Flagellin	Bacterial cell wall component	Invivogen	100ng/mL	TLR5
	R837	Small synthetic antiviral molecule	Invivogen	10ug/mL	TLR7
	R848	Small synthetic antiviral molecule	Invivogen	3ug/mL	TLR7/8
	CL097	Small synthetic antiviral molecule	Invivogen	5ug/mL	TLR7/8
	CpG 2006	Synthetic oligonucleotide	Invivogen	1uM	TLR9
	CpG 2216	Synthetic oligonucleotide	Invivogen	1uM	TLR9
	MDP	Bacterial cell wall component	Invivogen	10ug/mL	NOD2
	Poly I:C-LMW-Lyovec	Synthetic analog of dsRNA	Invivogen	1ug/mL	RIG-I
Cytokines	IL-1b	Recombinant Cytokine	BIIR	100ng/mL	IL1R
	IL-10	Recombinant Cytokine	Peprotech	100ng/mL	IL10R
	IL-15	Recombinant Cytokine	Peprotech	100ng/mL	IL15R
	IL-21	Recombinant Cytokine	Invitrogen	100ng/mL	IL21R
	TNFa	Recombinant Cytokine	R&D	50ng/mL	TNFAR
	IFNa	Recombinant Cytokine	Schering-Plough	500U/mL	IFNAR
Pathogens	Influenza H1N1 Brisbane	Virus	In house (BIIR)	MOI 5:1	TLR3/TLR7 ?
	Heat-killed *S. enterica*	Gram-negative bacteria	In house (BIIR)	10^8/mL	TLR4 / ?
	Heat-killed *S. aureus*	Gram-positive bacteria	Invivogen	10^8/mL	TLR2 / ?
